# Supplementary material for: Combinatorial design of molecular seeds for chirality-controlled synthesis of single-walled carbon nanotubes
Source: Nat Commun. 2019 Jul 22;10:3278. doi: 10.1038/s41467-019-11192-y (PMC6646389; doi:10.1038/s41467-019-11192-y)
Supplement: Supplementary file 1 — Supplementary Information [file 41467_2019_11192_MOESM1_ESM.pdf]

Supplementary Information for:

Combinatorial Design of Molecular Seeds for Chirality-Controlled Synthesis of Single-Walled Carbon Nanotubes

J. Tomada et al,

Table of content

Supplementary Methods 4
Experimental Part 5
1-bromo-4-methyl-naphthalene (S1) 5
1-bromo-4-(bromomethyl)naphthalene (S2) 5
2-(4-bromo-1-naphthyl)acetonitrile (S3) 6
2-(4-bromo-1-naphthyl)acetic acid (S4) 6
5-bromo-2H-acenaphthylen-1-one (S5) 7
5-bromo-1,2-dihydroacenaphthylen-1-ol (S6) 7
5-(9-anthryl)-1,2-dihydroacenaphthylen-1-ol (S7) 8
5-(9-anthryl)-*2H*-acenaphthylen-1-one (S8) 9
5-(2-phenylphenyl)-2H-acenaphthylen-1-one (S9) 10
2-([1,1'-biphenyl]-2-yl)-4,4,5,5-tetramethyl-1,3,2-dioxaborolan (S10) 10
1-([1,1'-biphenyl]-2-yl)-4-methylnaphthalen (S11) 11
6-methylbenzo[*g*]chrysene (S12) 11
6-(bromomethyl)benzo[*g*]chrysen (S13) 12
2-(benzo[*g*]chrysen-6-yl)acetonitrile (S14) 12
2-(benzo[*g*]chrysen-6-yl)acetic acid (S15) 13
Benzo[*g*]cyclopenta[*qr*]chrysen-4(5H)-on (S16) 13
2-methyldibenzo[*c,g*]chrysene (S18) 14
2-(bromomethyl)dibenzo[*c,g*]chrysene (S19) 15
2-(dibenzo[*c,g*]chrysen-2-yl)acetonitrile (S20) 15
2-(dibenzo[*c,g*]chrysen-2-yl)acetic acid (S21) 16
Benzo[*g*]indeno[1,7-*bc*]chrysen-9(*8H*)-one (S22) 16
Cyclotrimerization 17
General procedure 17
Trimers: NMR & HRMS Data 18
Alternative Combination Strategy 19
Cyclotetramerization 19
NMR-Data 20
MS-Data (MALDI-TOF) 36
Combination of the segments A and B 37

Combination of the segments A and C 40
Combination of the segments B and C 41
Cyclomerization of segment A 42
Cyclomerization of segment B 43
Crystallographic Data 44
Segment A - (S9) 44
Segment A´ - (S16) 45
Segment B - (S8) 46
Segment C - (S22) 47
UV/VIS Data 48
Precursors obtained in this work 489

Supplementary Methods

All chemicals were purchased from Sigma-Aldrich or ChemPUR and used without any further purification. Reactions that require an inert atmosphere were degassed by three cycles (3x 1 min) of sonication under membrane-pump vacuum, followed by the exchange of the atmosphere with nitrogen or argon.

Thin layer chromatography (TLC) was performed on Merck silica gel 60 F254, and visualized by *UV*-light (254 nm, 366 nm). Silica gel plug filtration was performed on Macherey–Nagel silica gel 60 M (230–400 mesh, 0.04–0.063 mm). HPLC analysis was carried out, using an analytical Cosmosil PBr (4.6 x 250 mm) column (Shimadzu, photo-diode array detector SPD-M20A). HPLC solvents were purchased from VWR.

NMR spectra were recorded on a Bruker Avance 400 or Jeol EX 400, operating at 400 MHz (^{1}H NMR) and 100 MHz (^{13}C NMR). Deuterated solvents were purchased from Sigma Aldrich and used as received. Signals were referenced to residual solvent peaks (δ in parts per million (ppm) $CDCl_3$: ^{1}H 7.24 ppm, ^{13}C 77.0 ppm; CD_2Cl_2: ^{1}H 5.32 ppm, ^{13}C 53.5 ppm. *o*-DCB-D4: ^{1}H 6.93 and 7.19 ppm, DMSO: ^{1}H 2.49 ppm. The resonance multiplicities are indicated as “s“ (singlet), “d” (doublet), “t” (triplet), and “m” (multiplet).

APPI mass spectra were recorded on a quadrupole time-of-flight (QqToF) mass spectrometer, the Bruker MAXIS. MALDI-TOF mass spectra were recorded with a Shimadzu Biotech Axima Confidence and a Bruker Reflex III.

STM experiments

The STM experiments were performed in ultrahigh vacuum conditions (base pressure 10^{-11} mbar) using a preparation chamber linked to a ScientaOmicron LT-STM/AFM operating at 5K. Platinum (111) crystals (MaTeck GmbH) were prepared by cycles of Ar+ sputtering (1keV) and thermal annealing (1100K), followed by a final flash above 1300K for 1 min. The precursor molecules were thermally evaporated (500°C) using a 6-fold organic evaporator (Mantis GmbH), while the substrate was held at room temperature (or higher as indicated). Images were acquired with ScientaOmicron’s Matrix software and subsequently analyzed with the MatrixFileReader XOP package (bytephysics) for Igor Pro (Wavemetrics).

Experimental Part

1-bromo-4-methyl-naphthalene (S1)

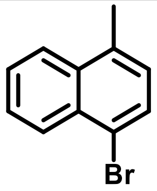

Chemical Formula: $C_{11}H_9Br$
Molecular Weight: 221,10

1-Methylnaphthaline (172 mL; 175 g; 1.23 mol) were solved in $CHCl_3$ (1 L). While cooling the solution in a water bath 63.1 mL of bromine (1.23 mol) were slowly added. The solution was then refluxed and monitored by TLC. After cooling the solution to room temperature, the solution was then twice washed with a NaOH solution (1M). Then the organic layer was dried over Na_2SO_4 and the solvent was evaporated. The resulting brown oil was then twice vacuum-distilled at 180 °C and 70 mbar, resulting in 247 g (1.12 mmol, 91 %) of the pure yellow oil. The measured ^{1}H- and ^{13}C-NMR corresponds to the reference compound.

1-bromo-4-(bromomethyl)naphthalene (S2)

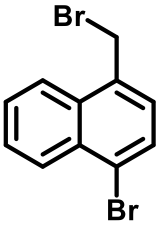

Chemical Formula: $C_{11}H_8Br_2$
Molecular Weight: 299,99

S2 was prepared by modified procedure from: M. Carreno et al., *J. Org. Chem.* 1999, *64*, 1387-1390.

5.0 g (23.0 mmol) of 1-bromo-4-methyl-naphthalene were dissolved in 30 mL of distilled DCM, then 4.5 g (25.3 mmol) and a catalytic amount of DBPO were added. The mixture was refluxed for 2 h (TLC monitoring). After cooling the solution to room temperature it was plugged through silica with hexane/DCM (2:1) as eluent. After evaporating the solvent the 5.1 g (17.0 mmol, 75 %) of the product were used without further purification in the next step. A small amount was purified for NMR analysis.

^{1}H-NMR corresponds to the spectra provided in the literature: V. V. Filichev, et al., *Angewandte Chemie, International Edition* 2006, *45*(32), 5311-5315).

R_f = 0.27 (SiO_2, Hex)

2-(4-bromo-1-naphthyl)acetonitrile (S3)

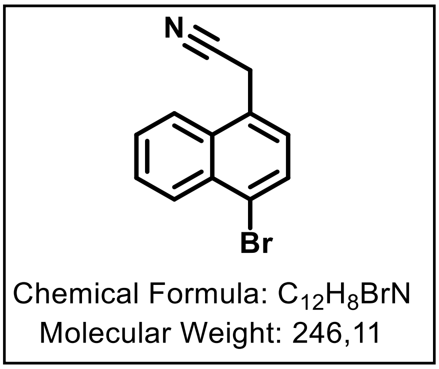

1.0 g (3,4 mmol) of 1-bromo-4-(bromomethyl) naphthalene were dissolved in 10.0 mL DCM, then a solution of 1.0 mL water and 437.2 mg (2 eq, 6.8 mmol) of KCN were added. After adding 65 mg of TBAB (5 mol %) the mixture was intensively stirred for 12 h at room temperature. After adding of 10 mL of water the solution was extracted with DCM (3x10 mL). The combined organic layers were dried over Na_2SO_4 and evaporated. Crude product was dissolved in DCM and plugged through silica gel. After evaporation732.0 mg (3.0 mmol, 89 %) pure **S3** was obtained as a white solid

R_f = 0.38 (SiO_2, Hex/DCM, 1:1).

^{1}H-NMR (400 MHz, $CDCl_3$) δ 8.31 (dd, *J* = 8.7, 0.8 Hz, 1H), 7.86 (ddd, *J* = 8.1, 3.2, 2.6 Hz, 2H), 7.72 – 7.53 (m, 3H), 4.10 (s, 2H).

^{13}C-NMR (101 MHz, $CDCL_3$) δ 134.12, 132.51, 128.80, 128.49, 128.38, 127.97, 127.46, 127.38, 126.28, 124.35, 117.30, 26.02);

HRMS (APPI): $[M]^+$ calculated for $C_{12}H_8BrN$ 244.9840; found 244.9835.

2-(4-bromo-1-naphthyl)acetic acid (S4)

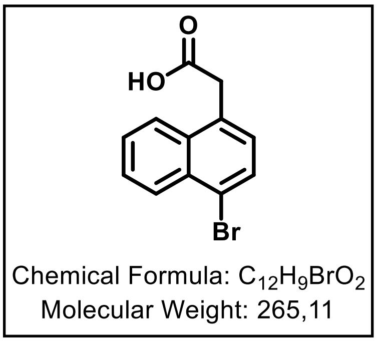

1.0 g (4.1 mmol) of **S3** were dissolved in 30 mL of acetic acid, then 10 mL of 10M sulfuric acid were slowly added under stirring. After leaving the solution for 12 h under reflux, the mixture was cooled to room temperature and diluted with 200 mL of water. After 2 h the white precipitate was filtered off, washed with water and dried under vacuum. 1.0 g (3.8 mmol, 96 %).

Note. The product can be obtained by direct bromination of 1-naphthaleneacetic acid in acetic acid (Thieme et al. , *Bioorganic & Medicinal Chemistry Letters* 2010, *20*(8), 2469-2473). However, in this case the final product contains about 10% of isomeric 2-(5-bromonaphthalen-1-yl) acetic acid) which is difficult to separate.

5-bromo-2H-acenaphthylen-1-one (S5)

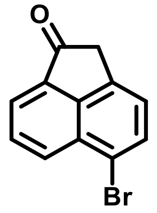

Chemical Formula: $C_{12}H_7BrO$
Molecular Weight: 247,09

1.0 g (3.8 mmol) of 2-(4-bromo-1-naphthyl) acetic acid were dissolved in 5 mL of thionylchloride and reflux for 90 min under nitrogen atmosphere. The excess of thionylchloride was removed under vacuum. The resulting oil was dissolved in 5 mL of DCM and cooled down to 0 °C (nitrogen atmosphere), before 1.0 g (2 eq, 7.6 mmol) of $AlCl_3$ was slowly added. The mixture was stirred for 1 h at 0 °C and then heated to reflux for 15 min. After cooling to room temperature, the mixture was slowly poured to a suspension of 100 g of ice and 10 mL of hydrochloric acid. After warming up to room temperature the product was extracted with DCM (3x50 mL). The combined organic layers were dried of Na_2SO_4 and the solvent was evaporated. Crude product was purified by flash column chromatography on silica, using hexane/ DCM (1:1) as an eluent. 608.0 mg (2.5 mmol, 65 %) of the desired ketone were obtained.

R_f = 0.39 (SiO_2, Hex/DCM, 1:1).

^{1}H-NMR (400 MHz, $CDCl_3$) δ 8.31 (dd, J = 8.7, 0.8 Hz, 1H), 7.86 (ddd, J = 8.1, 3.2, 2.6 Hz, 2H), 7.76 – 7.48 (m, 3H), 4.10 (s, 2H).

^{13}C-NMR (100 MHz,) δ 172.78, 133.57, 132.73, 131.54, 129.98, 129.07, 127.89, 127.52, 127.29, 125.28, 121.40, 38.43.

HRMS (APPI): $[M]^+$ calculated for $C_{12}H_7BrO$ 245.9680; found 245.9684.

5-bromo-1,2-dihydroacenaphthylen-1-ol (S6)

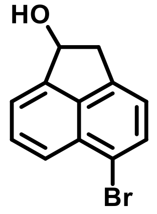

Chemical Formula: $C_{12}H_9BrO$
Molecular Weight: 249,11

1.0 g (4.0 mmol) of 5-bromo-2H-acenaphthylen-1-one were dissolved in 20 mL of a mixture of THF and methanol (1:1) and after adding of 230.0 mg (1.5 eq, 6.0 mmol) of $NaBH_4$ left for 2 h under stirring at room temperature. The solution was quenched with 50 mL of water and extracted three times with each 30 ml of toluene. The organic layer was then dried over Na_2SO_4 and evaporated resulting in pure **S6**. 988.0 mg (4.0 mmol, 98 %).

^{1}H NMR (400 MHz, $C_2D_2Cl_4$) δ 7.87 (d, J = 8.3 Hz, 1H), 7.67 (d, J = 7.3 Hz, 1H), 7.62 (dd, J = 8.2, 7.0 Hz, 1H), 7.54 (d, J = 7.0 Hz, 1H), 7.13 (d, J = 7.3 Hz, 1H), 5.67 (dd, J = 7.1, 2.2 Hz, 1H), 3.69 (ddd, J = 17.8, 7.1, 1.1 Hz, 1H), 3.12 (ddd, J = 17.9, 2.3, 1.3 Hz, 1H).

^{13}C NMR (100 MHz, $C_2D_2Cl_4$) δ 146.24, 141.91, 138.33, 131.74, 130.72, 129.77, 124.78 , 121.79, 121.23, 117.65, 41.68 (one signal overlaps).

HRMS (APPI): $[M]^+$ calculated for $C_{12}H_9BrO$ 247.9837; found 247.9841.

5-(9-anthryl)-1,2-dihydroacenaphthylen-1-ol (S7)

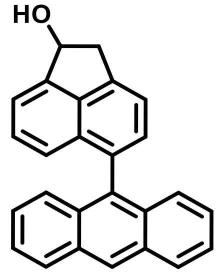

Chemical Formula: $C_{26}H_{18}O$
Molecular Weight: 346,43

1.0 g (4.0 mmol) 5-bromo-1,2-dihydroacenaphthylen-1-ol, 1.5 g (1.2eq, 4.9 mmol) 2-(9-anthryl)-4,4,5,5-tetramethyl-1,3,2-dioxaborolane and 2.6 g (2 eq, 8.0 mmol) Cs_2CO_3 were added to 40 mL of toluene/methanol solution (3:1). The solution was then degassed and (5 % mol) Pd(dppf)Cl_2 were added under a nitrogen atmosphere. The solution was refluxed for 12 h, cooled down to room temperature and diluted with 50 mL of water. The resulting mixture was extracted with toluene (3x20 mL). The organic layers were combined and dried over Na_2SO_4 and then filtrated through silica gel. After evaporation of toluene and the crude product was purified by flash column chromatography using DCM as an eluent. 941.0mg (2.6 mmol, 68 %).

^{1}H NMR (400 MHz, CD_2Cl_2) δ 8.52 (s, 1H), 8.03 (d, *J* = 8.6 Hz, 2H), 7.46 (d, *J* = 1.8 Hz, 2H), 7.41 – 7.31 (m, 4H), 7.25 – 7.13 (m, 3H), 6.79 (d, *J* = 8.3 Hz, 1H), 5.78 (t, *J* = 5.8 Hz, 1H), 3.99 - 3.80 (m, 1H), 3.32 (ddd, *J* = 17.7, 2.5, 0.7 Hz, 1H).

^{13}C NMR (100 MHz, CD_2Cl_2) δ 146.36 , 141.96 , 137.40 , 134.31 , 132.10 , 131.53 , 131.48 , 131.46 , 131.12 , 131.04 , 130.96 , 128.41 , 128.39 , 128.24 , 126.79 , 126.73 , 126.65 , 123.75 , 120.48 , 119.76 , 74.44 , 41.81 (four signals overlap).

HRMS (APPI): $[M]^+$ calculated for $C_{26}H_{18}O$ 346.1358; found 346.1357.

5-(9-anthryl)-*2H*-acenaphthylen-1-one (S8)

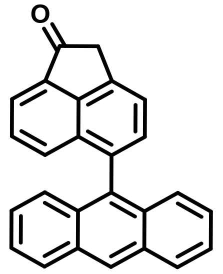

Chemical Formula: $C_{26}H_{16}O$
Molecular Weight: 344,41

Route a) 400 mg (1.2 mmol) of 5-(9-anthryl)-1,2-dihydroacenaphthylen-1-ol and 258,7mg (1eq, 1.2 mmol) PCC were dissolved in 10 mL of DCM and stirred at room temperature. The reaction was monitored by TLC, after no starting material was visible anymore the reaction was quenched with water and twice extracted with DCM. The combined organic layers were then dried over Na_2SO_4 and filtered through silica. After the solvent was evaporated the substance was separated by flash column chromatography (hexane/DCM, 1:1). 138.1 mg (0.5 mmol, 46 %).

Route b) 300 mg (1.2 mmol) of 5-bromo-2H-acenaphthylen-1-one, 300mg (1.1 eq, mmol) of anthraceneboronic acid and 240.7 mg (2.0 eq, 1.8 mmol) of K_2CO_3 were dissolved in 7.0 mL of freshly distilled THF and 3.5 mL of water (2:1) (nitrogen atmosphere). The mixture was three times degassed before $Pd(PPh_3)_4$ as a catalyst was added. After heating the reaction mixture under reflux for 24 h the solution was cooled to room temperature. After standard work-up, the crude product was purified by flash column chromatography (DCM/hexane, 2:1). 270.5mg (0.8 mmol, 64%).

R_f = 0.52 (SiO2, DCM/Hexane, 2:1).

^{1}H NMR (400 MHz, CD2Cl2) δ 8.52 (s, 1H), 8.02 (d, J = 8.5 Hz, 2H), 7.83 (d, J = 6.9 Hz, 1H), 7.57 (dd, J = 19.7, 7.0 Hz, 2H), 7.35 (m, 5H), 7.19 – 7.13 (m, 3H), 3.87 (s, 2H).

^{13}C NMR (100 MHz, CD2Cl2) δ 202.50, 143.08, 135.19, 133.62, 133.23, 131.46, 131.23, 131.01, 130.38, 129.99, 129.56 , 128.50, 128.20, 127.26, 126.48, 125.79, 125.25, 121.28, 121.07, 42.00 (three signals overlap).

HRMS (APPI): [M]+ calculated for C26H16O 344.1202; found 344.1201.

5-(2-phenylphenyl)-2H-acenaphthylen-1-one (S9)

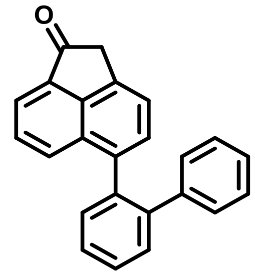

Chemical Formula: $C_{24}H_{16}O$
Molecular Weight: 320,39

1.0 g (4.0 mmol) of 5-bromo-2H-acenaphthylen-1-one, 1.4 g (1.2 eq, 5.0 mmol) of 4,4,5,5-tetramethyl-2-(2-phenylphenyl)-1,3,2-dioxaborolane and 2.0 g (2eq, 8.0 mmol) of Cs_2CO_3 were dissolved in 20 mL of toluene/methanol mixture (3/1). After degassing the solution and setting it under a nitrogen atmosphere, 5 mol % of $Pd(PPh_3)_4$ catalyst were added and the mixture was refluxed for 12 h. The work-up as described for S8. 930.5 mg (2.9 mmol, 72 %).

^{1}H NMR (400 MHz, CD_2Cl_2) δ 7.74 (dd, J = 7.0, 0.5 Hz, 1H), 7.71 (dd, J = 8.3, 0.7 Hz, 1H), 7.47 – 7.37 (m, 5H), 7.28 (s, 2H), 7.01 (ddd, J = 4.7, 2.3, 1.5 Hz, 2H), 6.96 – 6.92 (m, 3H), 3.67 (s, 2H).

^{13}C-NMR (101 MHz, CD_2Cl_2) δ 202.90, 143.14, 142.25, 141.81, 137.88, 137.27, 135.20, 134.69, 132.02, 130.86, 130.77, 130.51, 130.27, 129.65, 129.63, 128.52, 128.08, 127.65, 126.80, 121.18, 120.99, 42.15. (two signals overlap).

HRMS (APPI): $[M]^+$ calculated for $C_{24}H_{16}O$ 320.1201; found 320.1203.

2-([1,1'-biphenyl]-2-yl)-4,4,5,5-tetramethyl-1,3,2-dioxaborolan (S10)

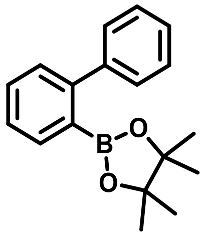

Chemical Formula: $C_{18}H_{21}BO_2$
Molecular Weight: 280,17

4,36 mL of 2-bromo-1,1'-biphenyl (25,3 mmol) and 8.36 g (pinacolato)diboron (1.3 eq, 32,9 mmol) were dissolved in 100 mL of dioxane. Then 4.97 g (2.0 eq, 50.6 mmol) of potassium acetate in 10 mL of water were added. The solution was degassed three times before the catalyst $Pd(dppf)Cl_2$ (5 mol%) was added (nitrogen atmosphere). The reaction mixture was refluxed for 12 h. The reaction mixture was then twice washed with 100 mL of water. The organic layer was dried over Na_2SO_4 and filtrated through silica (using DCM as an eluent). After removing the solvent the substance was separated by flash-chromatography (Hex:DCM, 3:1) yielding S10 as a white solid. 4.69 g (17.7 mmol, 66%) ^{1}H- and ^{13}C-NMR spectra were found to be in accordance with reported the literature (B. Singaram et al., *J. Org. Chem.* 2011, *76* (23), 9602–9610).

R_f=0.36 (SiO_2, Hex:DCM 2:1).

1-([1,1'-biphenyl]-2-yl)-4-methylnaphthalen (S11)

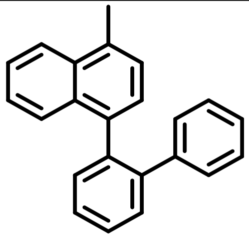

Chemical Formula: $C_{23}H_{18}$
Molecular Weight: 294,40

1,57 mL of S1 (10,1 mmol), 3,38 g of S10 (1.3 eq 12,1 mmol) und 2.80 g of K_2CO_3 (22,2 mmol) were dissolved in a 3:1 mixture of toluene/methanol. Under nitrogen atmosphere the solution was degassed before adding the catalyst $Pd(PPh_3)_4$ (5 mol%). The solution was heated under reflux for 12 h, before cooling it to room temperature and extracting it with 2x100 mL of water. The organic layer was dried over Na_2SO_4, before the solvent was removed. The crude product was purified by flash chromatography on silica (Hexane) resulting in 1.90 g (6.5 mmol, 64 %) of **S11** as a colourless oil.

R_f=0,27 (SiO_2, Hexane).

^{1}H NMR (400 MHz, $CDCl_3$) δ 7.97 (d; J=8.4 Hz; 1H); 7.70 (d; J=8.4 Hz; 1H); 7.54-7.39 (m; 5H); 7.34 (ddd; J=8.2; 6.8; 1.2 Hz; 1H); 7.17 (d; J=7.1 Hz; 1H); 7.08-7.01 (m; 6H); 2.66 (s; 3H).

^{13}C NMR (100 MHz, $CDCl_3$) δ 142.0; 141.6; 139.2; 137.7; 133.4; 132.6; 132.3; 132,.0; 130.3; 129.2; 127.9; 127.7; 127.6; 126.9; 126.9; 126.3; 125.9; 125.4; 125.3; 124.2; 19.4 (three signals overlap).

6-methylbenzo[*g*]chrysene (S12)

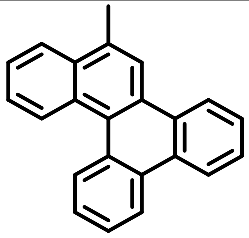

Chemical Formula: $C_{23}H_{16}$
Molecular Weight: 292,38

4.37 g (14.9 mmol) of S11, 4.15 g I_2 (1.1 eq, 16,3 mmol) und 10.4 mL propyleneoxide (10.0 eq, 149 mmol) were dissolved in 400 mL of cyclohexane and left at room temperature, under stirring in an UV-reactor (using a 400 Watt medium pressure mercury lamp). After competition (TLC monitoring) the solution was washed with sodium thiosulfate solution. The organic layer was dried over Na_2SO_4, before removing the solvent which resulted in 3.94 g (13.5 mmol, 90 %) of **S12**. The compound was used in the next step without further separation. A small amount of **S12** were purified by flash chromatography for characterisation.

R_f=0.27 (SiO_2, Hex).

^{1}H NMR (400 MHz, CD_2Cl_2) δ 8.95-8.92 (m; 1H); 8.85-8.83 (m; 1H); 8.76-8.66 (m; 3H) 8.48 (s; 1H); 8.21-8.18 (m; 1H); 7.75-7.62 (m; 6H); 2.90 (s; 3H).

HRMS (APPI): $[M]^+$ calculated for $C_{23}H_{16}$ 292.1252; found 292.1255.

6-(bromomethyl)benzo[*g*]chrysen (S13)

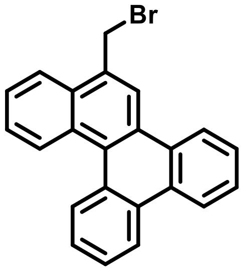

Chemical Formula: $C_{23}H_{15}Br$
Molecular Weight: 371,28

220 mg (0.752 mmol) 6-Methylbenzo[g]chrysen, 147 mg NBS (1.1 eq, 0.827 mmol) and 50 mg DBPO (0.207 mmol) were solved in 30ml CCl_4 and for 2 h refluxed. After cooling the solution to room temperature it was plugged through silica (washed with toluene), then the solvent was removed. The resulting 240 mg (0.647 mmol, 86 %) of the product were used without further purification in the next step.

R$_f$=0.26 (SiO_2, Hex:DCM 1:1).

2-(benzo[*g*]chrysen-6-yl)acetonitrile (S14)

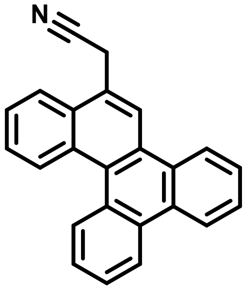

Chemical Formula: $C_{24}H_{15}N$
Molecular Weight: 317,39

175 mg (0.471 mmol) of 6-(bromomethyl)benzo[*g*]chrysen were dissolved in 20 mL of DCM and 2.5 eq (58.8 mg; 1.2 mmol) of NaCN in 5 mL water were added. Then 10 mg (0.030 mmol) of TBAB were added and the mixture was stirred at room temperature for 3 h. The suspension was then extracted with DCM (3x10 mL) before the combined organic layers were dried over Na_2SO_4. The crude product was purified by flash chromatography on silica (Hexane:DCM 1:1). 140 mg (0.442, mmol, 94 %)

R$_f$=0.25 (Hex:DCM 1:1).

^{1}H NMR (400 MHz, CD_2Cl_2) δ 9.01-8.97 (m; 1H); 8.83 (d; J=8.2 Hz; 1H); 8.79-8.69 (m; 4H); 8.09-8.05 (m; 1H); 7.79-7.66 (m; 6H); 4.36 (d; J=0.8 Hz, 2H).

^{13}C NMR (100 MHz, $CDCl_3$) δ 131.6; 131.3; 131.1; 130.5; 130.0; 129.9; 129.6; 129.3; 128.2; 128.1; 128.1; 127.7; 127.6; 127.3; 126.9; 126.8; 125.9; 124.1; 124.0; 123.7; 123.2; 122.1; 118.3; 22.7.

HRMS (APPI): $[M]^+$ calculated for $C_{24}H_{15}N$ 317.1204; found 292.1210.

2-(benzo[*g*]chrysen-6-yl)acetic acid (S15)

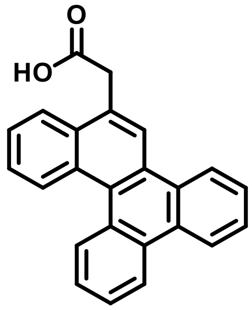

Chemical Formula: $C_{24}H_{16}O_2$
Molecular Weight: 336,39

79.0 mg (0.249 mmol) of 2-(benzo[*g*]chrysen-6-yl)acetonitrile were dissolved in 20 mL of acetic acid. Then a mixture of 2 mL water and 2 mL H_2SO_4 was added and the solution was refluxed for 12 h. After cooling down to room temperature 400 mL of water were added to the solution. The resulting suspension was left for two h before the precipitate was filtered off and washed with water resulting in 83 mg (0.247 mmol, 98 %).

^{1}H-NMR (400 MHz, DMSO-d6) δ 8.92-8.78 (m; 6H); 8.16-8.13 (m; 1H); 7.81-7.69 (m; 6H); 4.32 (s; 2H); 3.50 (br s; 1H).

^{13}C NMR (100 MHz, DMSO-D6) δ173.0; 132.3; 131.7; 130.4; 129.9; 129.6; 129.0; 128.5; 128.5; 127.9; 127.8; 127.2; 127.1; 126.8; 126.5; 126.2; 125.9; 124.5; 124.1; 124.0; 123.6; 123.3 (the signals of methylene carbon overlaps with the DMSO signal).

Benzo[*g*]cyclopenta[*qr*]chrysen-4(5H)-on (S16)

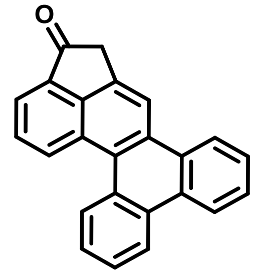

Chemical Formula: $C_{24}H_{14}O$
Molecular Weight: 318,3750

62.0 mg (0.184 mmol) of S15 were dissolved in 30.0 mL of DCM and 0.2 mL of DMF (2.60 mmol) under nitrogen atmosphere. The reaction mixture was cooled down 0°C and 0.6 mL (888 mg, 7,0 mmol) $C_2O_2Cl_2$ were slowly added. After refluxing for one h, the solvent was evaporated and resulting oil was dissolved in 30 mL of DCM. The solution was cooled to 0°C under nitrogen atmosphere and 49.2 mg (0.369 mmol) of $AlCl_3$ were slowly added in small portions. Then the solution was stirred for one h at 0°C before getting refluxed for 15 min. The reaction mixture was then cooled down to room temperature and poured to a suspension of 100 g ice containing 10 mL of concentrated hydrochloric acid. The mixture was then twice extracted with 100 mL of DCM. The organic layers were combined and dried over Na_2SO_4. The solvent was removed and the resulting crude product was purified by flash chromatography using toluene as eluent, resulting in S16 as yellow solid. 46 mg (0.147 mmol ,78 %).

R_f=0,53 (Hex:DCM 1:2).

^{1}H-NMR (400 MHz, CD_2Cl_2) δ9.17 (d; J=8.3 Hz; 1H); 8.99-8.95 (m; 1H); 8.81-8.70 (m; 3H); 8.67 (s; 1H); 8.04 (d; J=7.0 Hz; 1H); 7.89 (dd; J=8.3; 7.1 Hz; 1H); 7.78-7.71 (m; 4H); 3.96 (s; 2H).

^{13}C NMR (100 MHz, CD_2Cl_2) δ202.8; 144.0; 135.5; 135.0; 133.2; 130.8; 130.6; 130.5; 130.4; 129.8; 128.9; 128.8; 128.7; 128.0; 127.9; 127.3; 127.2; 125.4; 124.4; 123.9; 123.7; 121.6; 117.0; 42.1.

HRMS (APPI): $[M]^+$ calculated for $C_{24}H_{14}O$ 318.1039; found 318.1034.

2-methyldibenzo[*c,g*]chrysene (S18)

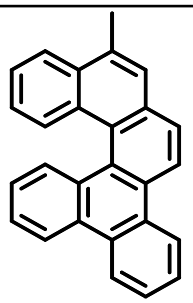

Chemical Formula: $C_{27}H_{18}$
Molecular Weight: 342,44

The starting 2-(2-methylstyryl)triphenylene was obtained according described in the literature protocol (A. Mueller, K. Yu. Amsharov, *Eur. J. Org. Chem*. 2015**,** *14,* 3053-3056). 3.1 g (9.0 mmol) of 2-(2-methylstyryl)triphenylene, 2.3 g of I_2 (1.1 eq, 9,1 mmol) und 5.0 mL of propyleneoxide (75 mmol) were dissolved in 600 mL of cyclohexane and left for 6 h at room temperature, under stirring in an UV-reactor (using a 400 Watt medium pressure mercury lamp). Then the solution was washed with sodium thiosulfate solution. The organic layer was dried over Na_2SO_4, before removing the solvent which resulted in 2.5 g (7.3 mmol, 83 %) of yellow solid . HPLC analysis show three products. Pure **S18** was obtained from the mixture after several recrystallizations from hot toluene.

$\mathbf{R_f}$ = 0.34 (SiO2, DCM/Hex, 1:5).

^{1}H-NMR (400 MHz, CD_2Cl_2) δ 8.75 – 8.68 (m, 1H), 8.66 – 8.57 (m, 3H), 8.46 – 8.42 (m, 1H), 8.30 – 8.25 (m, 1H), 8.11 (dd, *J* = 8.3, 1.2 Hz, 1H), 7.92 (d, *J* = 8.5 Hz, 1H), 7.74 (dd, *J* = 6.7, 2.8 Hz, 3H), 7.55 (dddd, *J* = 8.2, 6.9, 2.5, 1.3 Hz, 2H), 7.22 (tdd, *J* = 8.3, 6.9, 1.3 Hz, 2H), 2.84 (d, *J* = 1.1 Hz, 2H).

^{13}C-NMR (100 MHz, CD_2Cl_2) δ 132.05, 132.04, 131.20, 131.09, 130.94, 130.80, 130.60, 130.55, 130.38, 130.14, 128.14, 128.06, 128.02, 127.89, 127.70, 127.65, 127.49, 127.24, 125.82, 125.78, 125.47, 124.51, 124.15, 123.74, 123.09, 122.60, 118.30, 22.50 (one signal overlaps).

HRMS (APPI): $[M]^+$ calculated for $C_{27}H_{18}$ 342.1409; found 342.1411.

2-(bromomethyl)dibenzo[*c,g*]chrysene (S19)

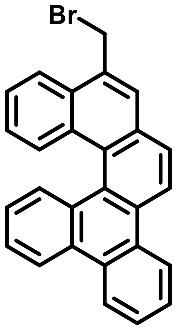

Chemical Formula: $C_{27}H_{17}Br$
Molecular Weight: 421,34

350 mg (1.023 mmol) of S18, 200 mg of NBS (1.1 eq, 1.125 mmol) and 50 mg of DBPO (0.207 mmol) were dissolved in 30 mL of CCl_4 and the mixture was refluxed for 2 h (TLC monitoring). After cooling the solution to room temperature it was plugged through silica (washed with toluene), and evaporated. The resulting 267 mg (0.634 mol, 62 %) of the S19 were used without further purification in the next step.

2-(dibenzo[*c,g*]chrysen-2-yl)acetonitrile (S20)

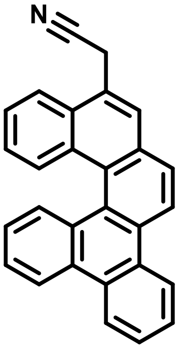

Chemical Formula: $C_{28}H_{17}N$
Molecular Weight: 367,45

250 mg (0.594 mmol) of S19 were solved in 20 mL of DCM and 97 mg (2.5 eq, 1.5 mmol) of KCN in 5 mL of water were added. Then 10 mg (0.030 mmol) TBAB were added and the mixture was intensively stirred at room temperature for 3 h. The suspension was then extracted with DCM (3x10mL) before the combined organic layers were dried over Na_2SO_4. The crude product was purified by flash chromatography (hexane/DCM 1:1) resulting in 187 mg (0.510 mmol, 86 %).

^{1}H-NMR (400 MHz, CD_2Cl_2) δ 8.62 (dd, *J* = 6.2, 3.3 Hz, 1H), 8.55 – 8.50 (m, 3H), 8.39 (d, *J* = 8.5 Hz, 1H), 8.14 (d, *J* = 8.3 Hz, 1H), 7.91 – 7.82 (m, 3H), 7.68 – 7.63 (m, 2H), 7.53 – 7.45 (m, 2H), 7.21 – 7.09 (m, 2H), 4.19 (s, 2H).

^{13}C-NMR (101 MHz, CD_2Cl_2) δ 132.05, 132.04, 131.20, 131.09, 130.94, 130.80, 130.60, 130.55, 130.38, 130.14, 128.14, 128.06, 128.02, 127.89, 127.70, 127.65, 127.49, 127.24, 125.82, 125.78, 125.47, 124.51, 124.15, 123.74, 123.09, 122.60, 118.30, 22.50.

HRMS (APPI): $[M]^+$ calculated for $C_{28}H_{17}N$ 367.1361; found 367.1364.

2-(dibenzo[*c,g*]chrysen-2-yl)acetic acid (S21)

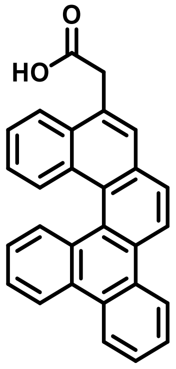

Chemical Formula: $C_{28}H_{18}O_2$
Molecular Weight: 386,45

0.7 g (1.9 mmol) of S20 were dissolved in 30 mL of acetic acid and 10 mL of 10M H_2SO_4 were added. After leaving the solution for 12 h under reflux, then cooling it to room temperature the solution was added to 200ml of water and leading to white precipitation. The suspension was left for 2 h. The precipitate was filtered off, washed with water and dried under vacuum. 692 mg (1.8 mmol), yield: 94 %. The compound was used in the next step as obtained.

Benzo[*g*]indeno[1,7-*bc*]chrysen-9(*8H*)-one (S22)

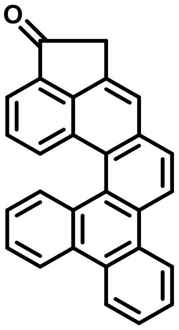

Chemical Formula: $C_{28}H_{16}O$
Molecular Weight: 368,44

10.0 mg (26 µmol) of S21 and one drop of DMF were added to 5mL of DCM under nitrogen atmosphere and the reaction mixture was cooled to 0°C. After adding 0.1 mL of $C_2O_2Cl_2$ the reaction mixture was heated under reflux for one h. After cooling the solution to room temperature and evaporating the solvent, the resulting oil was dissolved in 5mL of DCM and cooled down to 0°C, before 7.1 mg (2eq, 52 µmol) of $AlCl_3$ were slowly added in small portions. After stirring of the reaction mixture at 0°C for one h. The solution was refluxed for 15 min and cooled to room temperature and poured into 10 g of ice containing 1 mL of concentrated HCl. After warming up to room temperature the reaction mixture was extracted with DCM (3x10 mL). The combined organic layers were dried of Na_2SO_4 and the solvent was evaporated. After a purification by flash column chromatography (toluene), 6.5 mg (18 µmol, 68 %) of the ketone **S22** were obtained.

^{1}H-NMR (400 MHz, CD_2Cl) δ 8.77 – 8.70 (m, 1H), 8.65 (dd, *J* = 7.7, 3.5 Hz, 4H), 8.53 (d, *J* = 8.3 Hz, 1H), 8.05 (d, *J* = 8.6 Hz, 1H), 7.95 (d, *J* = 7.1 Hz, 1H), 7.86 (s), 7.76 (dd, *J* = 5.9, 3.6 Hz, 2H), 7.63 (dd, *J* = 15.2, 1.2 Hz, 1H), 7.48 (dd, *J* = 8.2, 7.2 Hz, 1H), 7.34 – 7.26 (m, 1H), 3.99 (d, *J* = 22.9 Hz, 1H), 3.85 (d, *J* = 21.8 Hz, 1H).

^{13}C-NMR (100 MHz,) δ 201.92 , 142.89 , 134.21 , 133.70 , 133.41 , 132.19 , 129.65 , 129.56 , 129.37 , 129.21 , 129.13 , 128.99 , 128.85 , 127.86 , 125.93 , 124.67 , 123.23 , 123.12 , 122.58 , 121.34 , 121.22 , 121.17 , 40.63 (one signal overlaps)

HRMS (APPI): $[M]^+$ calculated for $C_{28}H_{16}O$ 368.1201; found 368.1203.

Cyclotrimerization

General procedure
(combinatorial assembly of trimers)

50 mg (0.05-0.06 mmol) of the corresponding ketone (homotrimer synthesis) or mixture of two ketones in 1:1 or 1:2 ratio (combinatorial assembly) were dissolved in 1,5 mL of *o*-DCB directly in a glass ampule. After adding of 0,1 mL (0,94 mmol) of $TiCl_4$ under argon atmosphere, the reaction mixture was freeze, degassed and the ampule was sealed under vacuum. The ampule was heated for 48 h at 150 °C. After cooling to room temperature the ampule was open and the reaction mixture was slowly poured into 20 mL of acetone and left for several h. The precipitate containing di- and trimers was filtered off (filtrate contains higher macrocycles and oligomers). The precipitate was dissolved in toluene and plugged through silica (toluene) to remove polar side products. Fraction containing trimers (MS analysis) was used further for HPLC separation. The overall yield of trimers was around 40-70% depending on segments and ratio used. The segments used and yields are summarized in Supplementary Fig 1 and Supplementary Table 1.

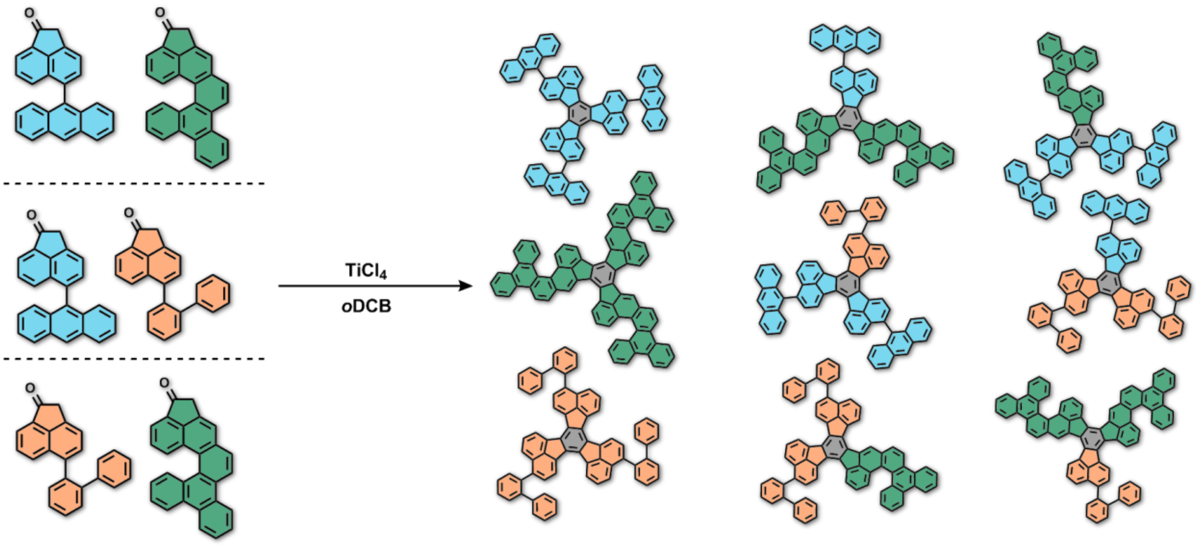

Supplementary Figure 1. Trimerization using the combinatorial approach.

Supplementary Table 1. Yields of the trimers in comparison to the segment ratio(determined by HPLC)

Ratio of A and B	1:1	1:2
**AAA**	41 %	15 %
**AAB**	20 %	30 %
**ABB**	29 %	35 %
**BBB**	10 %	20 %

Trimers: NMR & HRMS Data

AAA ^{1}H NMR (400 MHz,) δ 8.62 – 8.36 (m, 4H), 7.68 – 7.33 (m, 22H), 7.22 – 7.07 (m, 7H), 7.07 – 6.90 (m, 9H).**HRMS** (LDI-MS) $[M]^+$ calculated for $C_{72}H_{42}$ 906.3287 found 906.3294.

AAB ^{1}H NMR (400 MHz, CD_2Cl_2) δ 8.96 – 8.78 (m, 1H), 8.77 – 8.63 (m, 3H), 8.60 (s, 1H), 8.13 – 7.96 (m, 2H), 7.83 – 7.74 (m, 1H), 7.73 – 7.37 (m, 22H), 7.32 – 7.19 (m, 2H), 7.18 – 7.11 (m, 4H), 7.08 – 6.93 (m, 6H). **HRMS** (LDI-MS): $[M]^+$ calculated for $C_{74}H_{42}$ 930.3287 found 930.3277.

ABB ^{1}H NMR (400 MHz, CD_2Cl_2) δ 8.12 (d, *J* = 7.0 Hz, 2H), 7.79 – 7.64 (m, 4H), 7.53 – 7.32 (m, 32H), 7.23 (ddd, *J* = 8.0, 7.1, 2.0 Hz, 4H). **HRMS** (LDI-MS): $[M]^+$ calculated for $C_{76}H_{42}$ 954.3287; found 954.3312.

BBB ^{1}H NMR (400 MHz, CD_2Cl_2) δ 8.39 – 8.02 (m, 6H), 7.84 (s, 3H), 7.33 (d, *J* = 8.5 Hz, 6H), 7.09 (d, *J* = 7.4 Hz, 3H), 6.81 (dd, *J* = 12.7, 6.8 Hz, 9H), 6.72 – 6.65 (m, 6H), 6.55 – 6.48 (m, 6H), 6.42 (d, *J* = 8.4 Hz, 3H) **HRMS** (LDI-MS): $[M]^+$ calculated for $C_{78}H_{42}$ 978.3287; found 978.3296.

AAC -2H **HRMS** (LDI-MS): $[M]^+$ calculated for $C_{76}H_{40}$ 952.3125; found 952.3123.

BBC -2H **HRMS** (LDI-MS): $[M]^+$ calculated for $C_{80}H_{40}$ 1000.3100; found 1000.3108.

AAAA HRMS (LDI-MS): $[M]^+$ calculated for $C_{96}H_{56}$ 1208.4382; found 1208.4373

AAAB HRMS (LDI-MS): $[M]^+$ calculated for $C_{98}H_{56}$ 1232.4382; found 1232.4371

A2B2 HRMS (LDI-MS): $[M]^+$ calculated for $C_{100}H_{56}$ 1256.4382; found 1256.4368

ABBB HRMS (LDI-MS): $[M]^+$ calculated for $C_{102}H_{56}$ 1280.4382; found 1280.4365

BBBB HRMS (LDI-MS): $[M]^+$ calculated for $C_{104}H_{56}$ 1304.4382; found 1304.4366

Alternative Combination Strategy

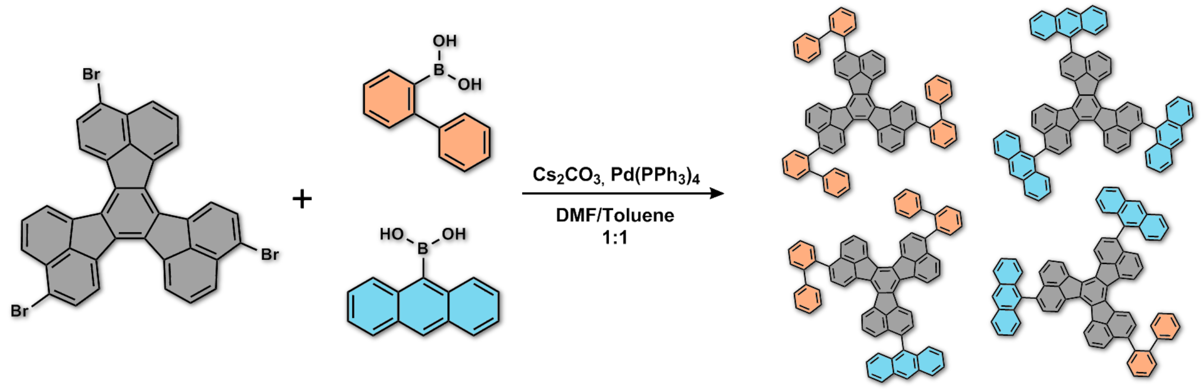

Supplementary Figure 2. Combinatorial Suzuki coupling.

As an alternative to the cyclotrimerisation of cyclic ketones, a route based on Suzuki coupling was tested.

89mg (0.12 mmol) tribromodecacyclen, and 13mg (0.5 eq, 0.06mmol) biphenyl-boronic acid and 43mg (1.5 eq, 0.18mmol) anthracene boronic acid were dissolved in 36 mL of DMF/toluene (2:1) mixture. The respective tribromodecacyclen was obtained through a cyclotrimerisation of S5 according general trimerization procedure. The reaction mixture was three degassed and heated for 9 h under nitrogen at 80°C. After cooling the solution to room temperature 50 mL water were added and the solution was extracted with toluene. The organic layers were combined and dried over Na_2SO_4 and then filtrated through silica gel. The following HPLC analysis showed the formation of the desired products along formation of several sideproducts, which were identified as mono- and bis- functionalized decacyclenes. Since the separation of differently substituted decacyclens appears to be very difficult, no further effort was done to improve the reaction conditions.

Cyclotetramerization

The tetramers were obtained using previously described protocol (Sumy et al., *Chem. Eur. J.* 2016, *22(*14), 4709-4712). Briefly: The mixture of TiCl4 (1 mL) in ODCB (10 mL) was heated to reflux under nitrogen atmosphere. Cyclic ketone (1mmol) dissolved in ODCB was added dropwise to the refluxing mixture. After consumption of the educt the hot reaction mixture was slowly poured into crushed ice / HCl mixture and extracted with DCM. Due to our experience with the trimerization for the tetramerization of A and B a 1:2 ratio was chosen. Further tetramerizations for the other combinations were carried out in a 1:1 ratio. All tetramerization (homo- and heterotetramers) were carried out in a 20 mg scale.

NMR-Data

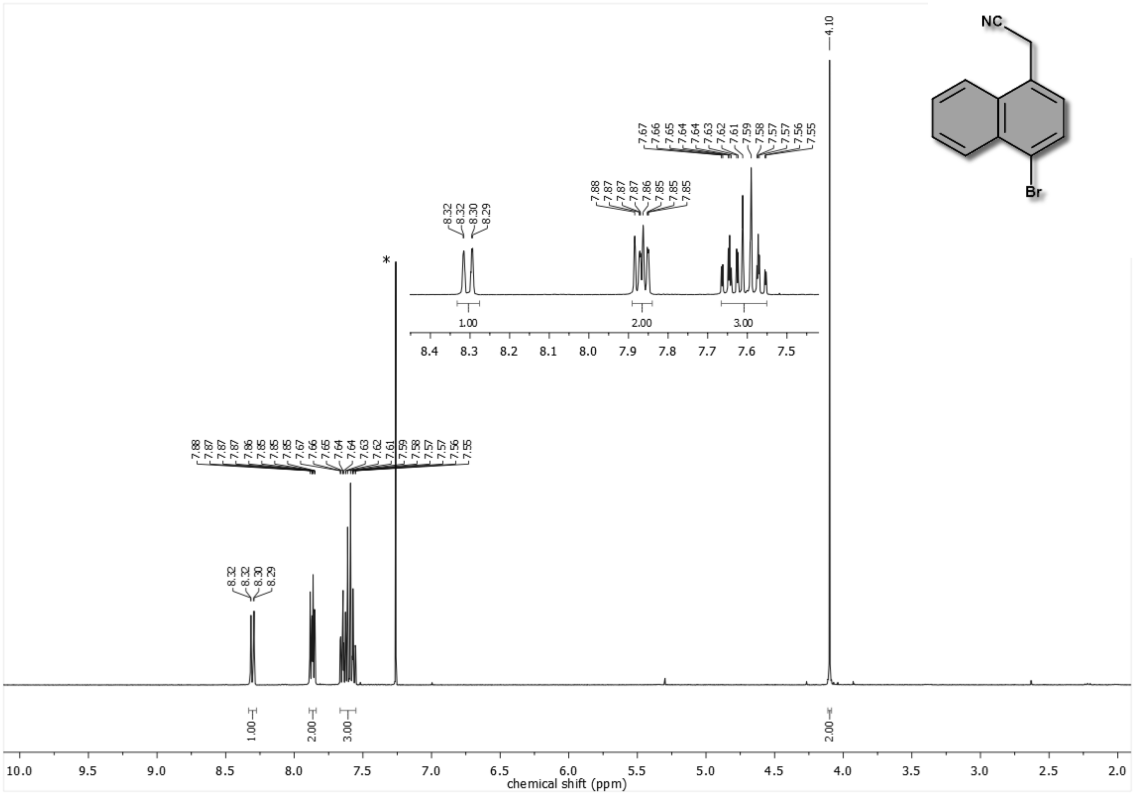

Supplementary Figure 3. 400MHz ^{1}H-NMR of 1-bromo-4-(bromomethyl)naphthalene in $CDCl_3$

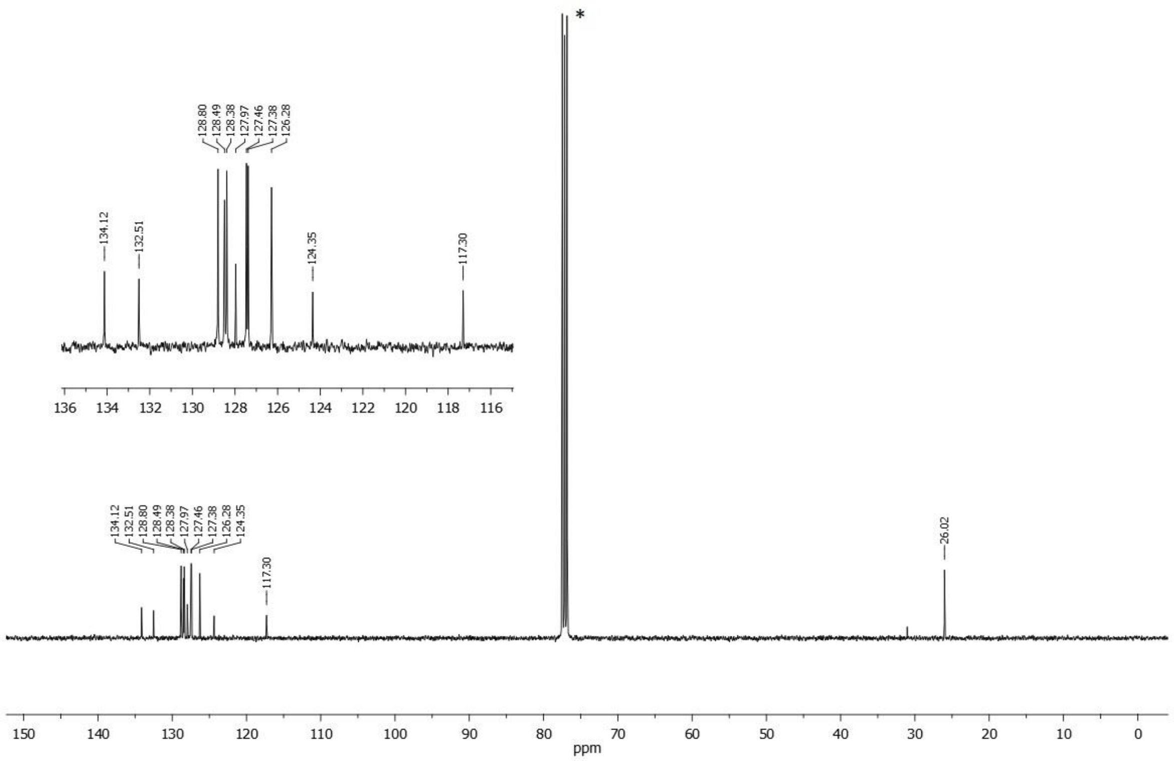

Supplementary Figure 4. 100MHz ^{13}C-NMR of 1-bromo-4-(bromomethyl)naphthalene in $CDCl_3$

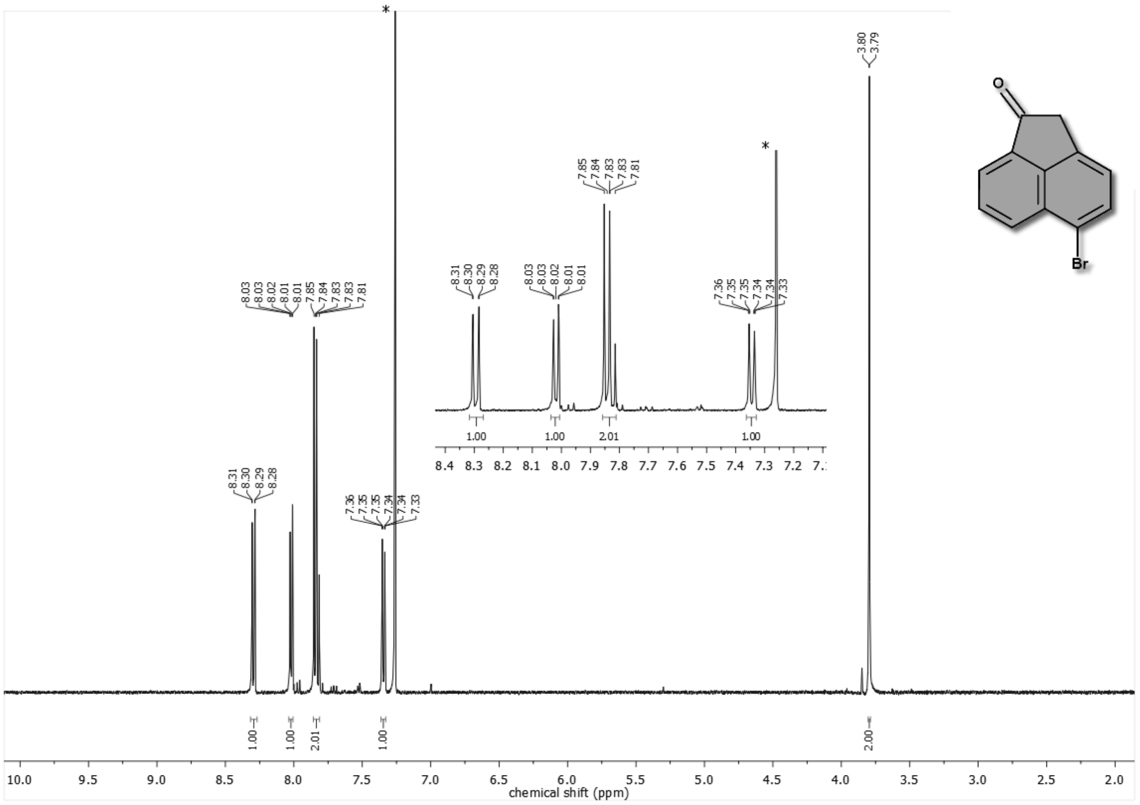

Supplementary Figure 5. 400 MHz ^{1}H NMR spectra of 5-bromo-2H-acenaphthylen-1-one in $CDCl_3$

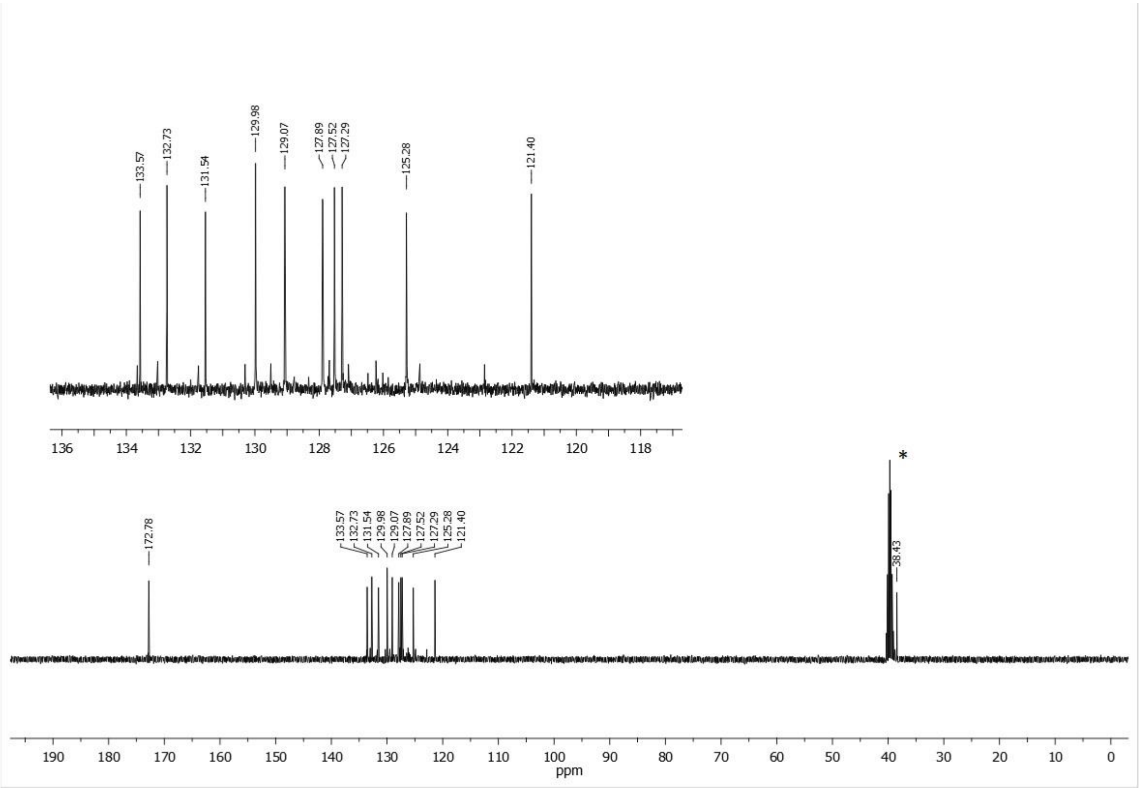

Supplementary Figure 6. 100 MHz ^{13}C NMR spectra of 5-bromo-2H-acenaphthylen-1-one in DMSO

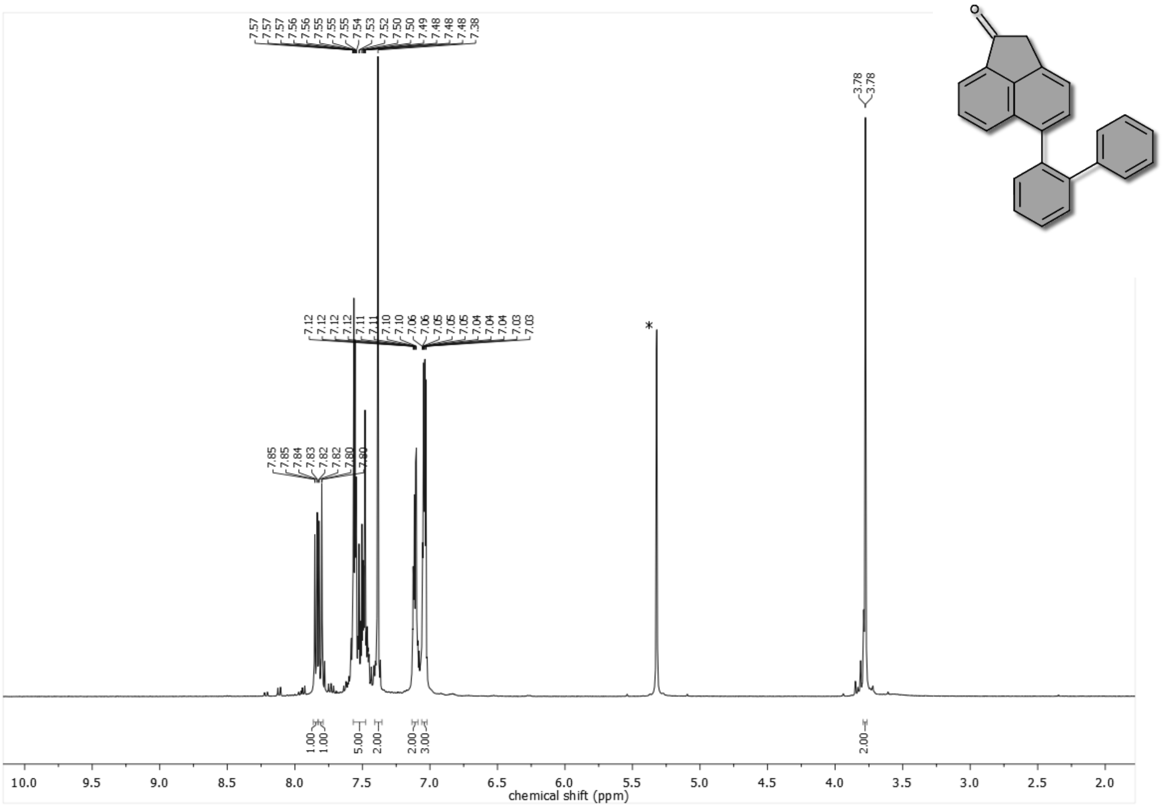

Supplementary Figure 7. ^{1}H NMR spectra of 5-(2-phenylphenyl)-2H-acenaphthylen-1-one in $CDCl_3$

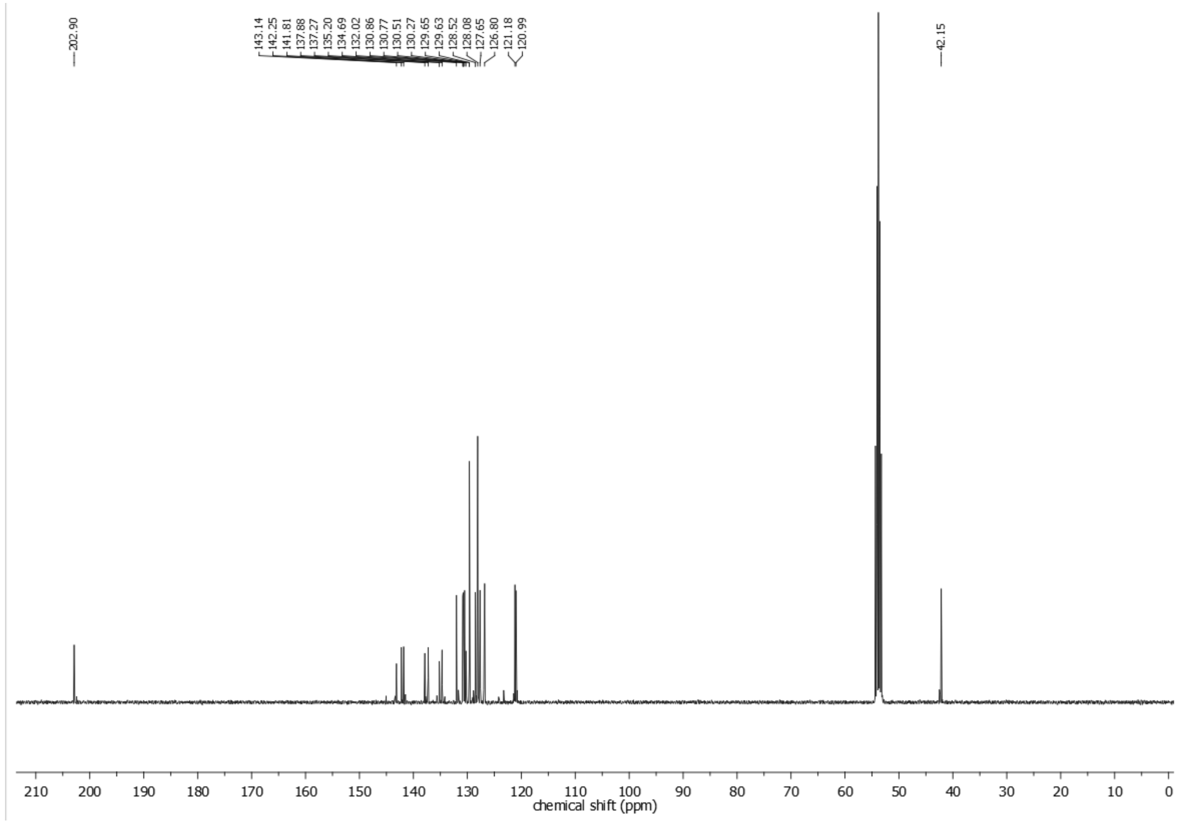

Supplementary Figure 8. ^{13}C NMR spectra of 5-(2-phenylphenyl)-2H-acenaphthylen-1-one in $CDCl_3$

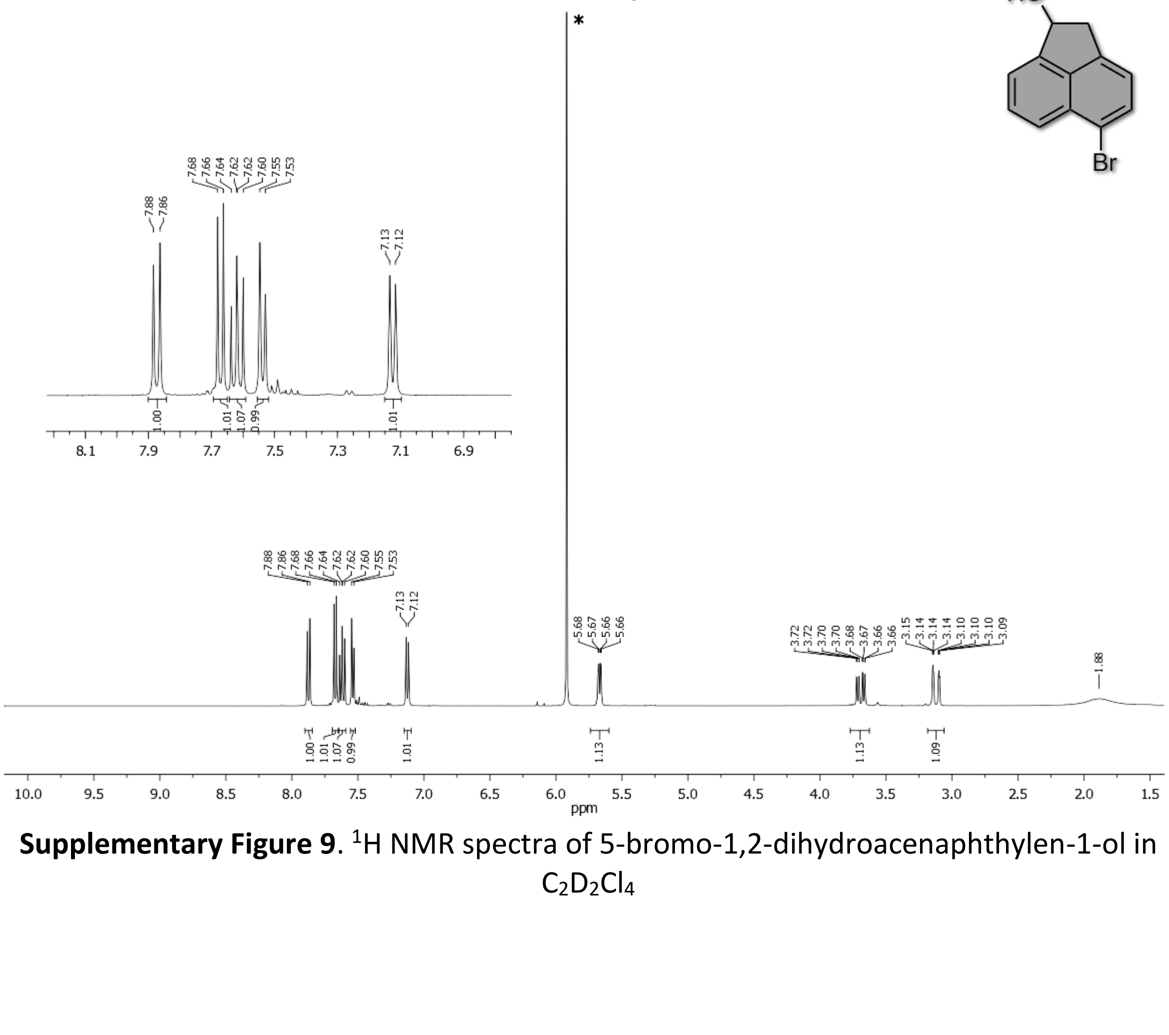

Supplementary Figure 9. ^{1}H NMR spectra of 5-bromo-1,2-dihydroacenaphthylen-1-ol in $C_2D_2Cl_4$

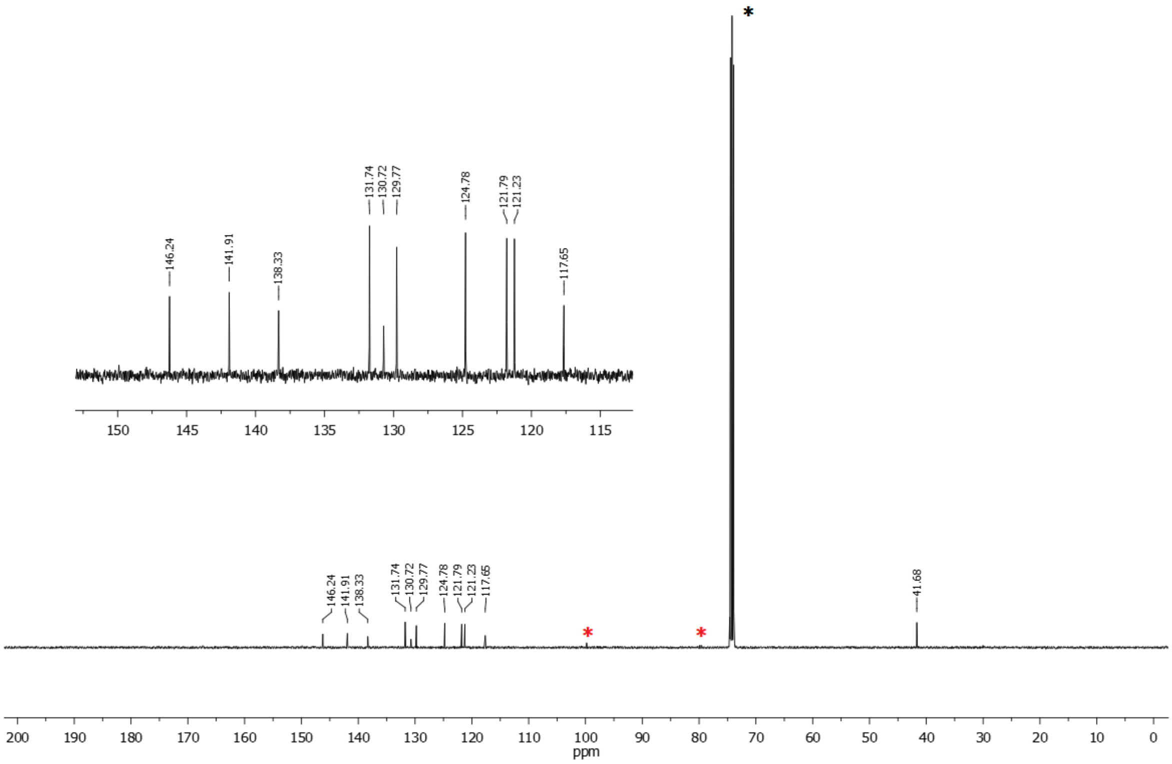

Supplementary Figure 10. ^{13}C NMR spectra of 5-bromo-1,2-dihydroacenaphthylen-1-ol in $C_2D_2Cl_4$

*solvent impurities

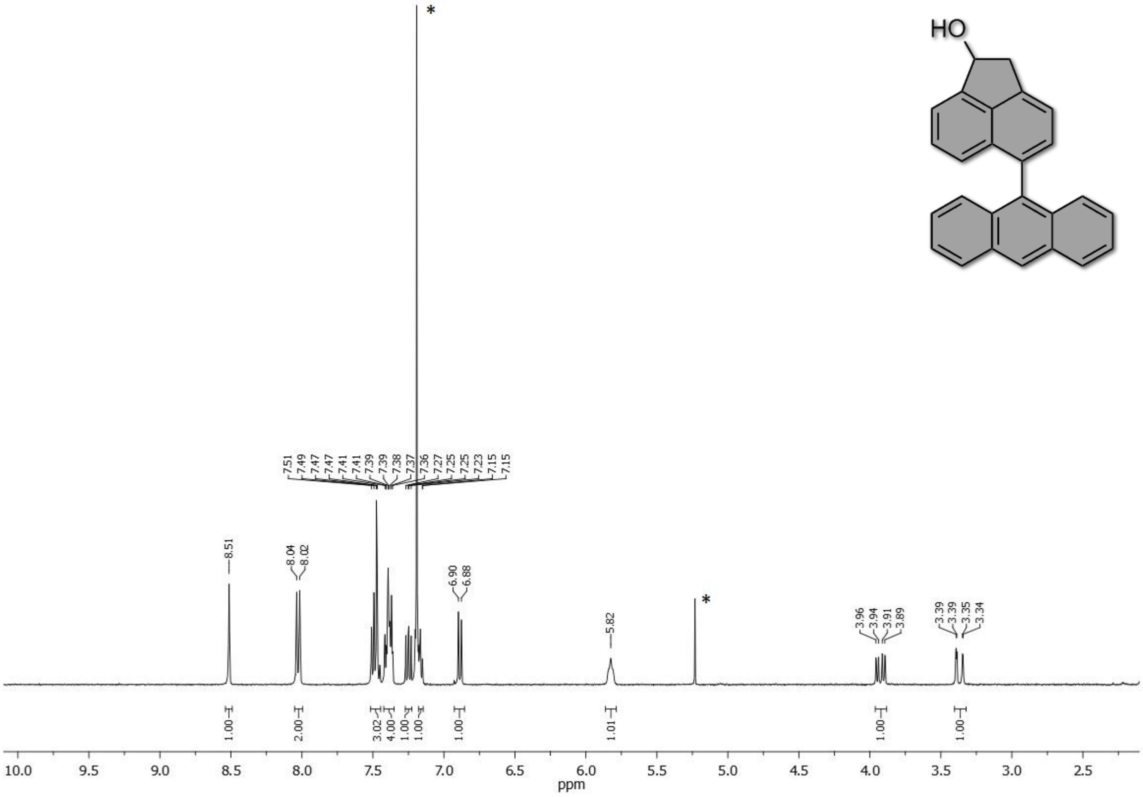

Supplementary Figure 11. 400MHz ^{1}H-NMR of 5-(9-anthryl)-1,2-dihydroacenaphthylen-1-ol in $CDCl_3$

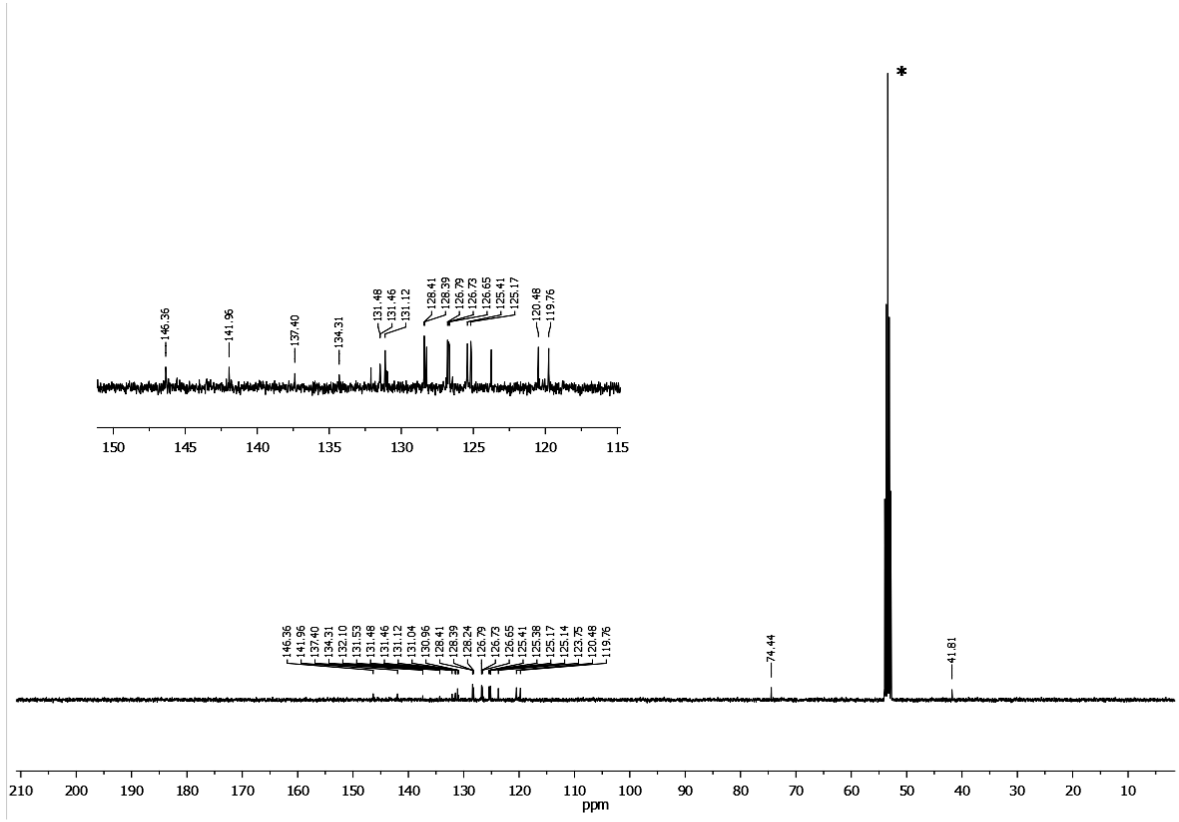

Supplementary Figure 12. ^{13}C NMR spectra of 5-(9-anthryl)-1,2-dihydroacenaphthylen-1-ol in CD_2Cl_2

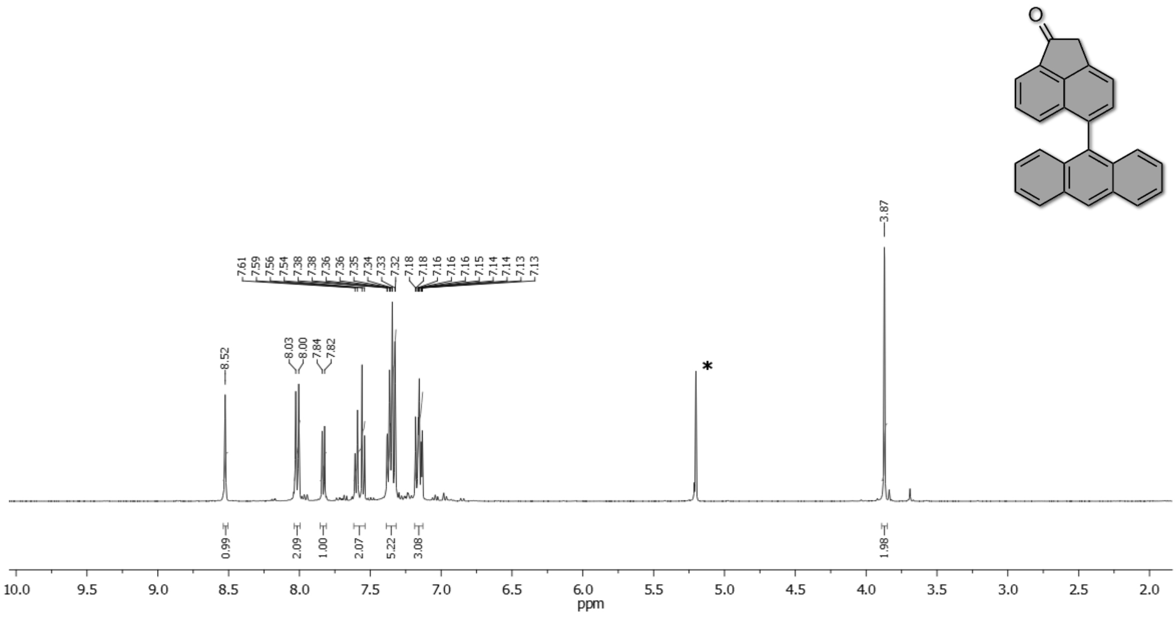

Supplementary Figure 13. 400MHz ^{1}H-NMR of 5-(9-anthryl)-2H-acenaphthylen-1-one in CD_2Cl_2

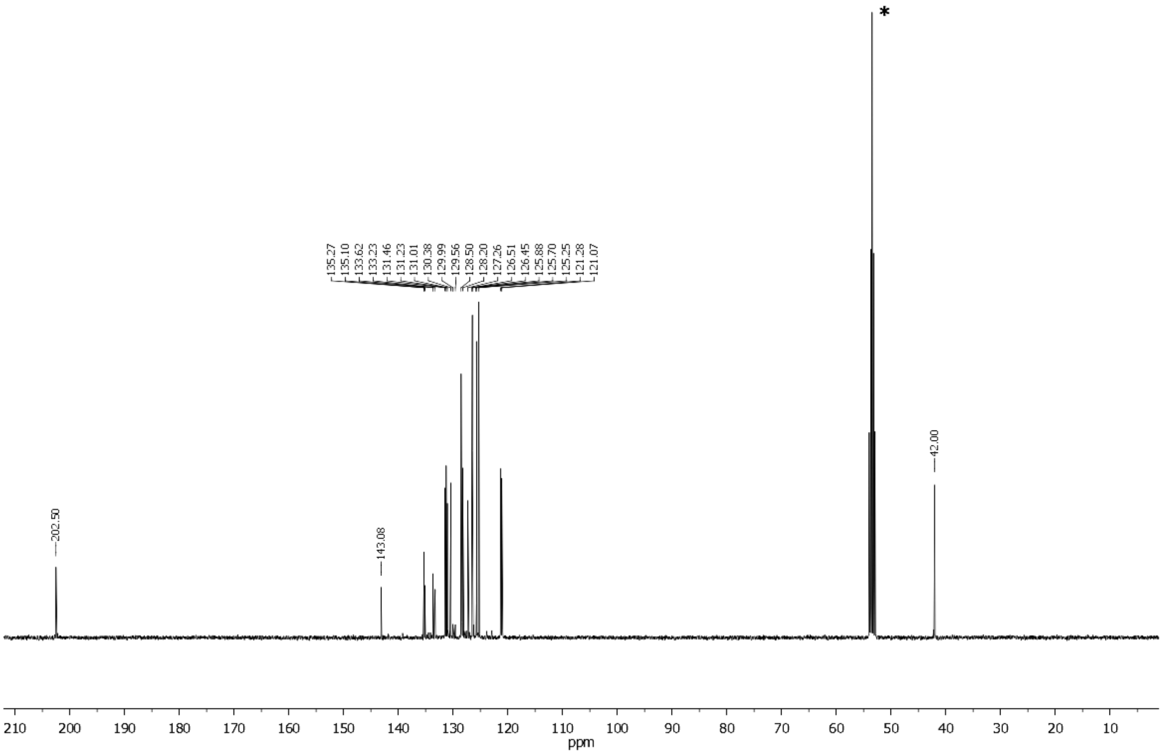

Supplementary Figure 13. ^{13}C NMR spectra of 5-(9-anthryl)-2H-acenaphthylen-1-one in CD_2Cl_2

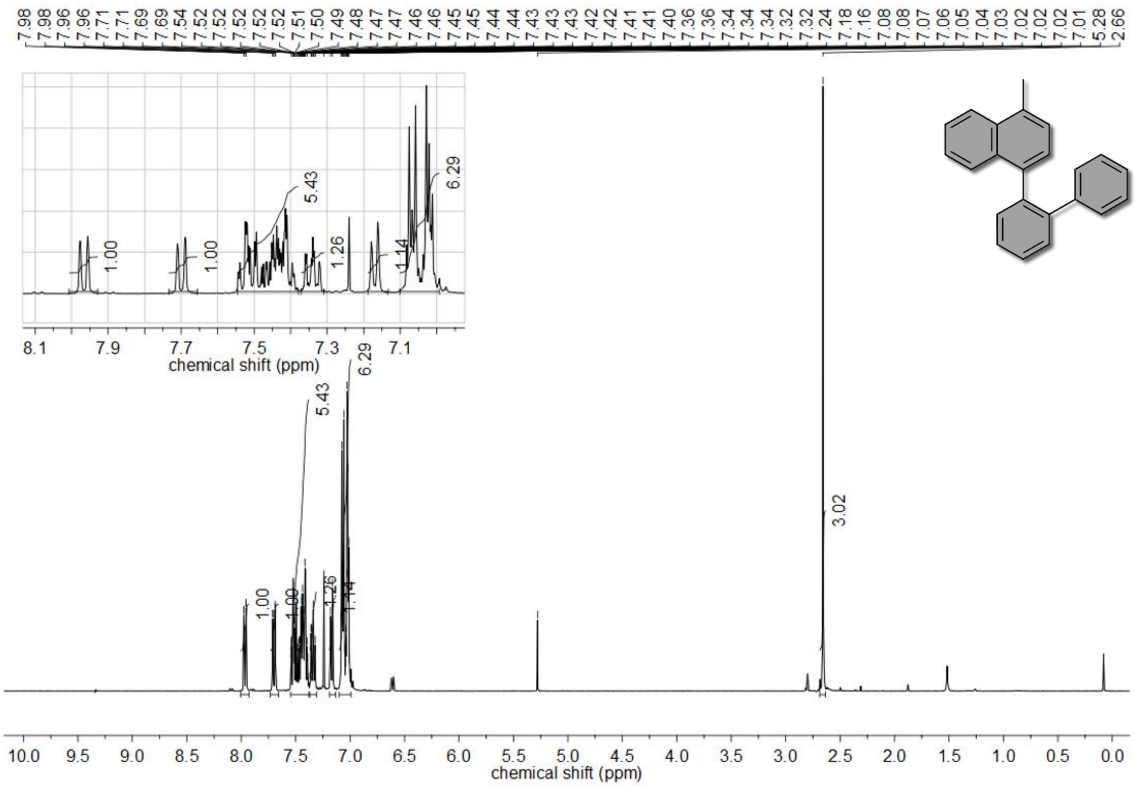

Supplementary Figure 14. 400 MHz ^{1}H NMR spectra of 1-methyl-4-(2-phenylphenyl)naphthalene in $CDCl_3$

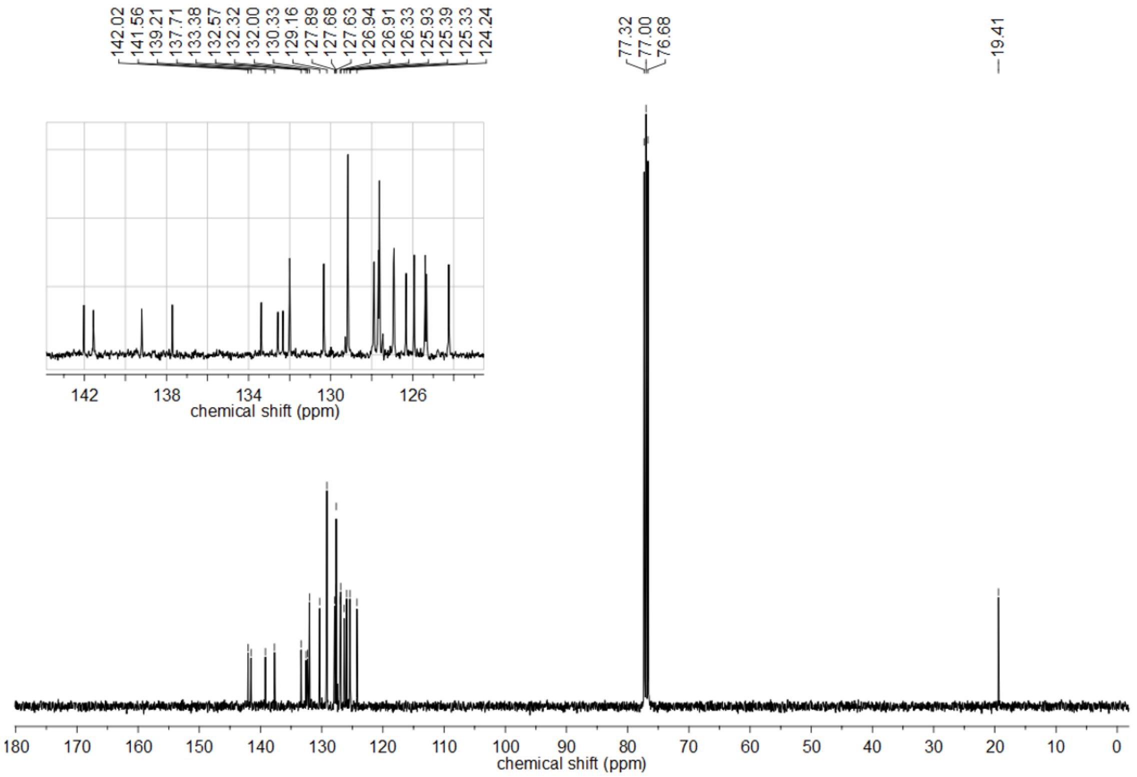

Supplementary Figure 15. 100 MHz ^{13}C NMR spectra of 1-methyl-4-(2-phenylphenyl)naphthalene in $CDCl_3$

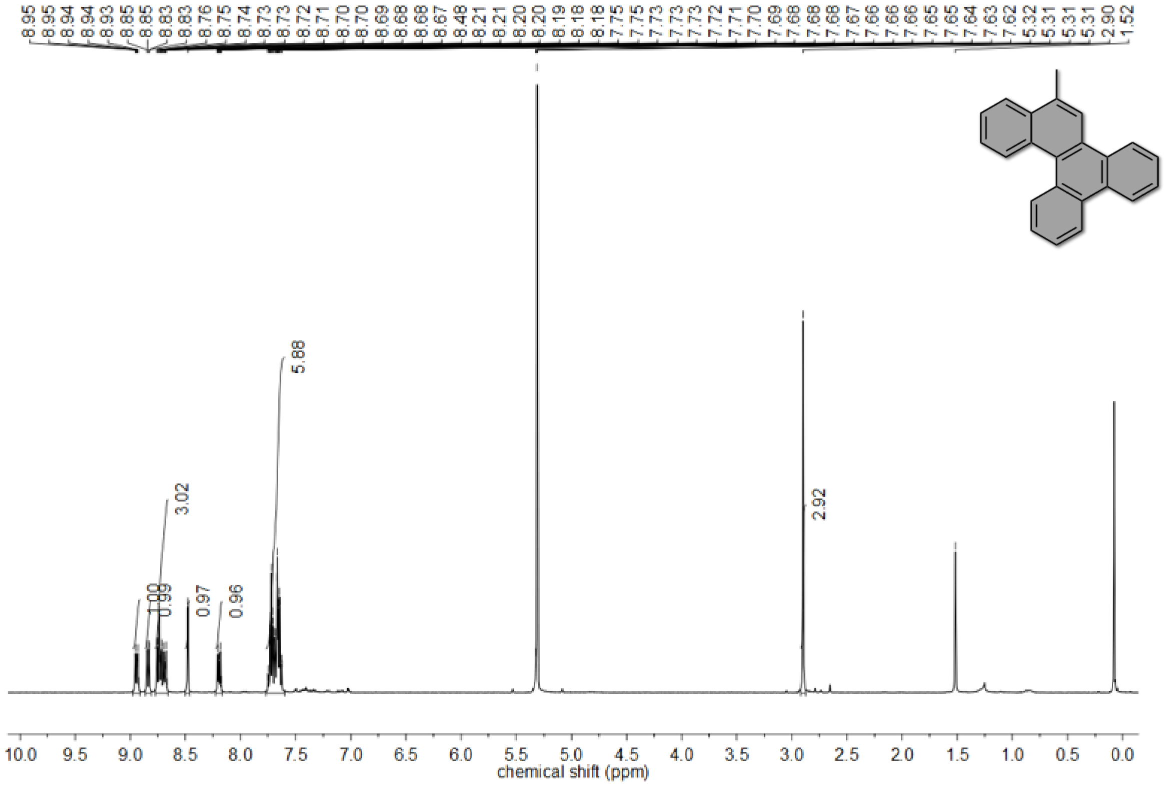

Supplementary Figure 16. 400 MHz ^{1}H NMR spectra of 6-methylbenzo[g]chrysene in CD_2Cl_2

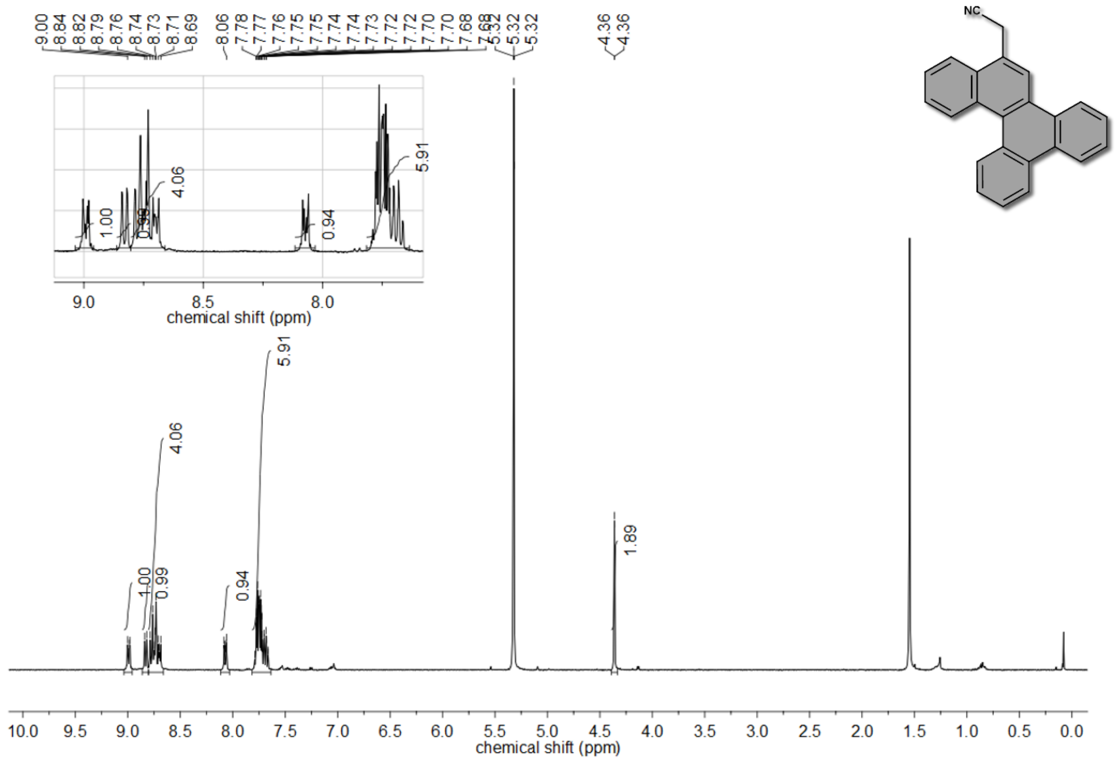

Supplementary Figure 17. 400MHz ^{1}H-NMR of 2-(Benzo[g]chrysen-6-yl)acetonitrile in CD_2Cl_2

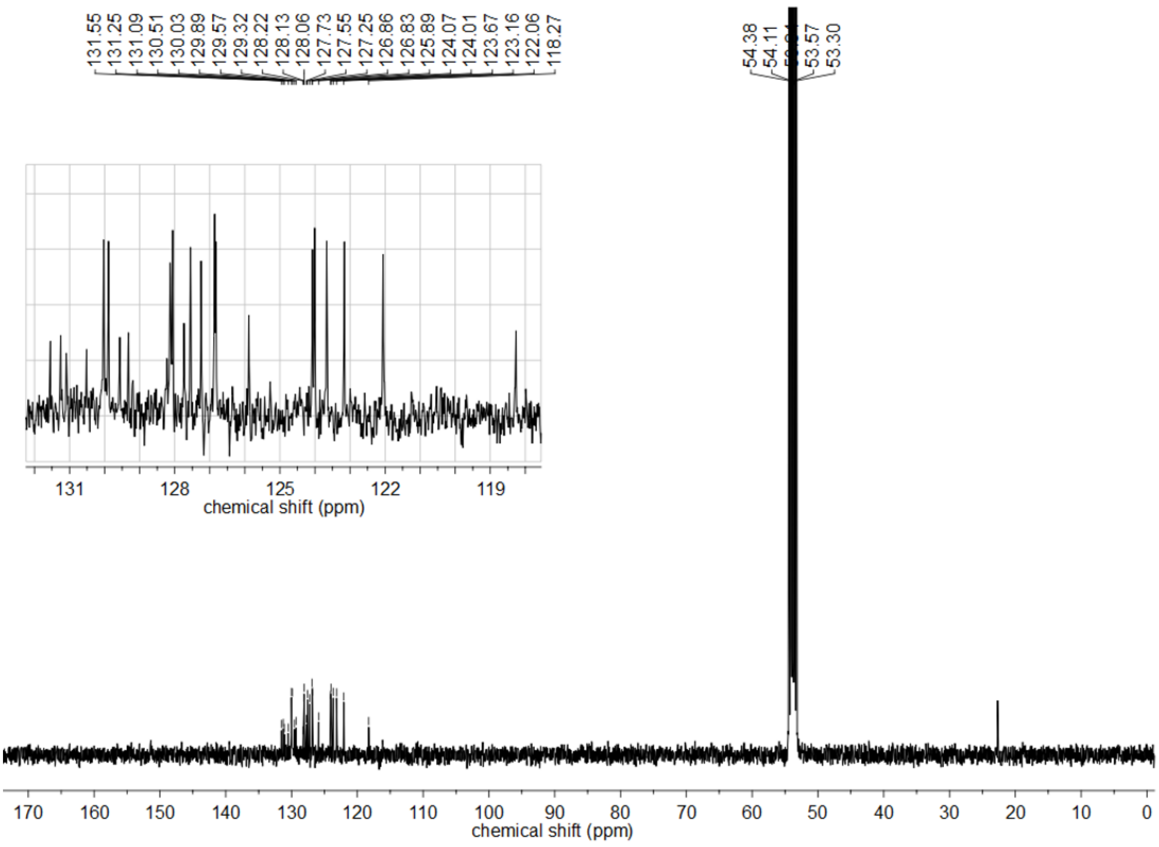

Supplementary Figure 18. 100MHz ^{13}C-NMR of 2-(Benzo[g]chrysen-6-yl)acetonitrile in CD_2Cl_2

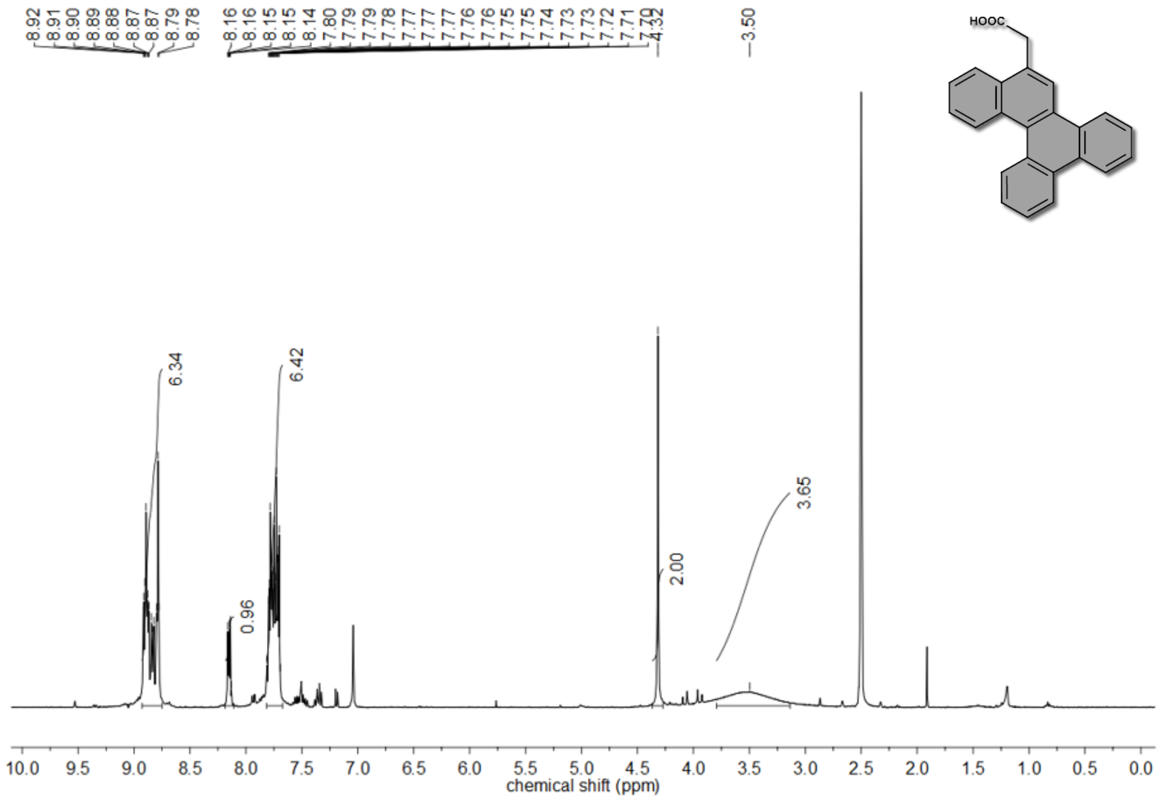

Supplementary Figure 19. 400 MHz ^{1}H-NMR of 2-(benzo[g]chrysen-6-yl)acetic acid in DMSO-d6

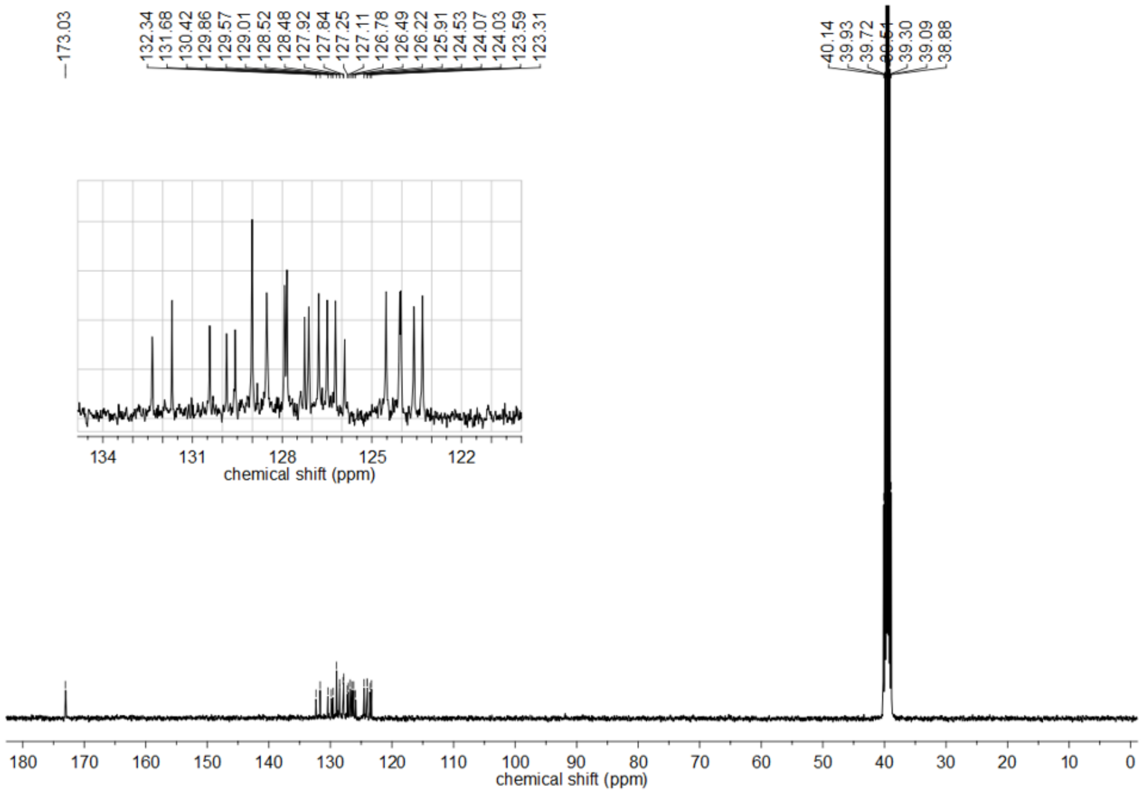

Supplementary Figure 20. 100 MHz ^{13}C-NMR of 2-(benzo[g]chrysen-6-yl)acetic acid in DMSO-d6

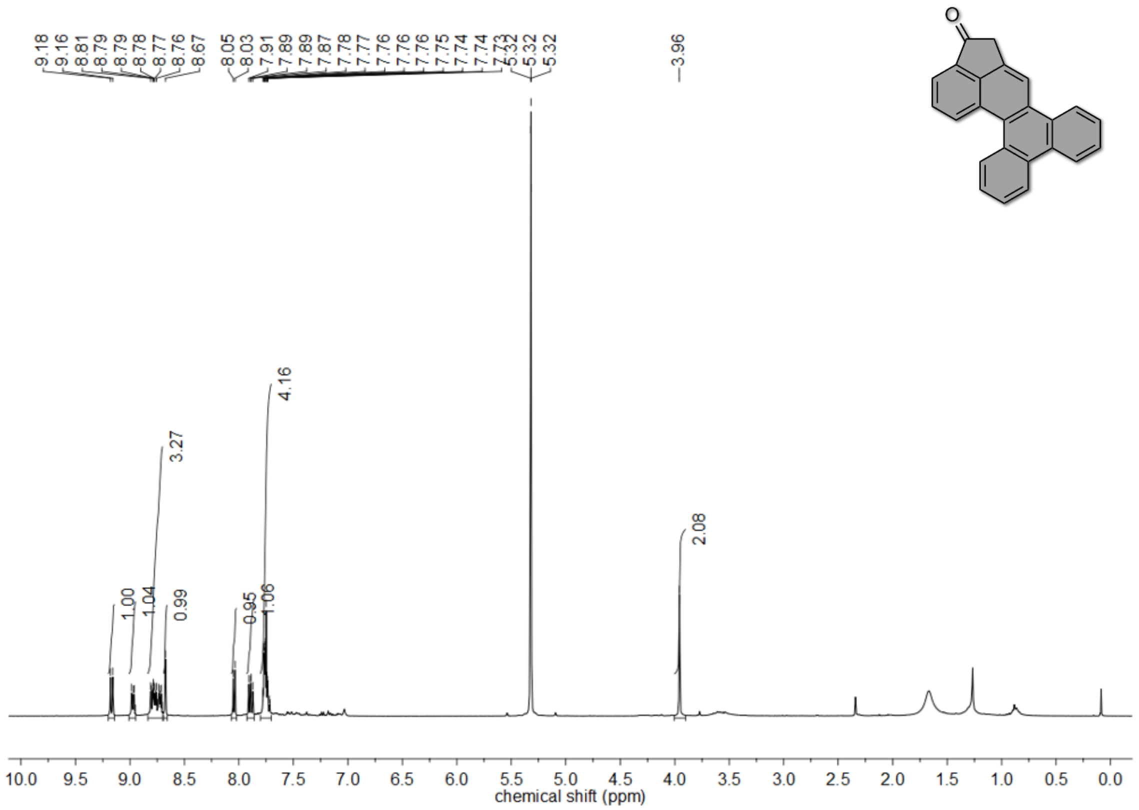

Supplementary Figure 21. ^{1}H-400 MHz Benzo[g]cyclopenta[qr]chrysen-4(5H)-on in CD_2Cl_2

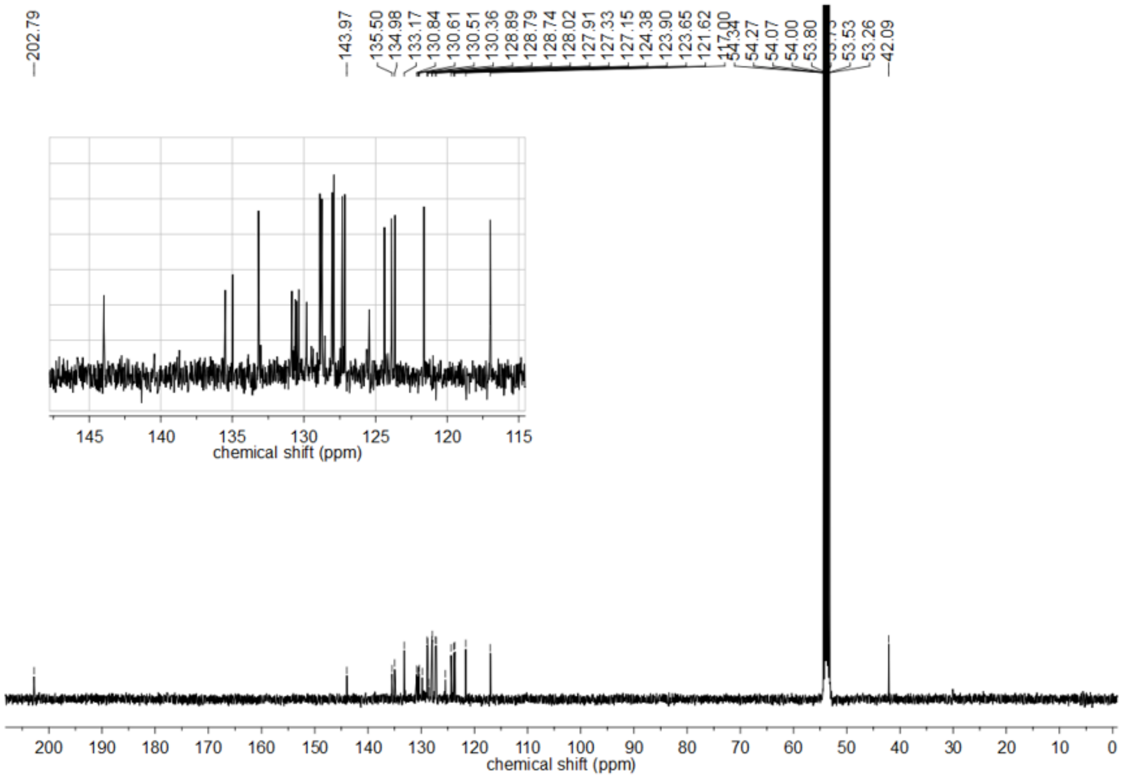

Supplementary Figure 22. 100 MHz ^{13}C-NMR of Benzo[g]cyclopenta[qr]chrysen-4(5H)-on in CD_2Cl_2

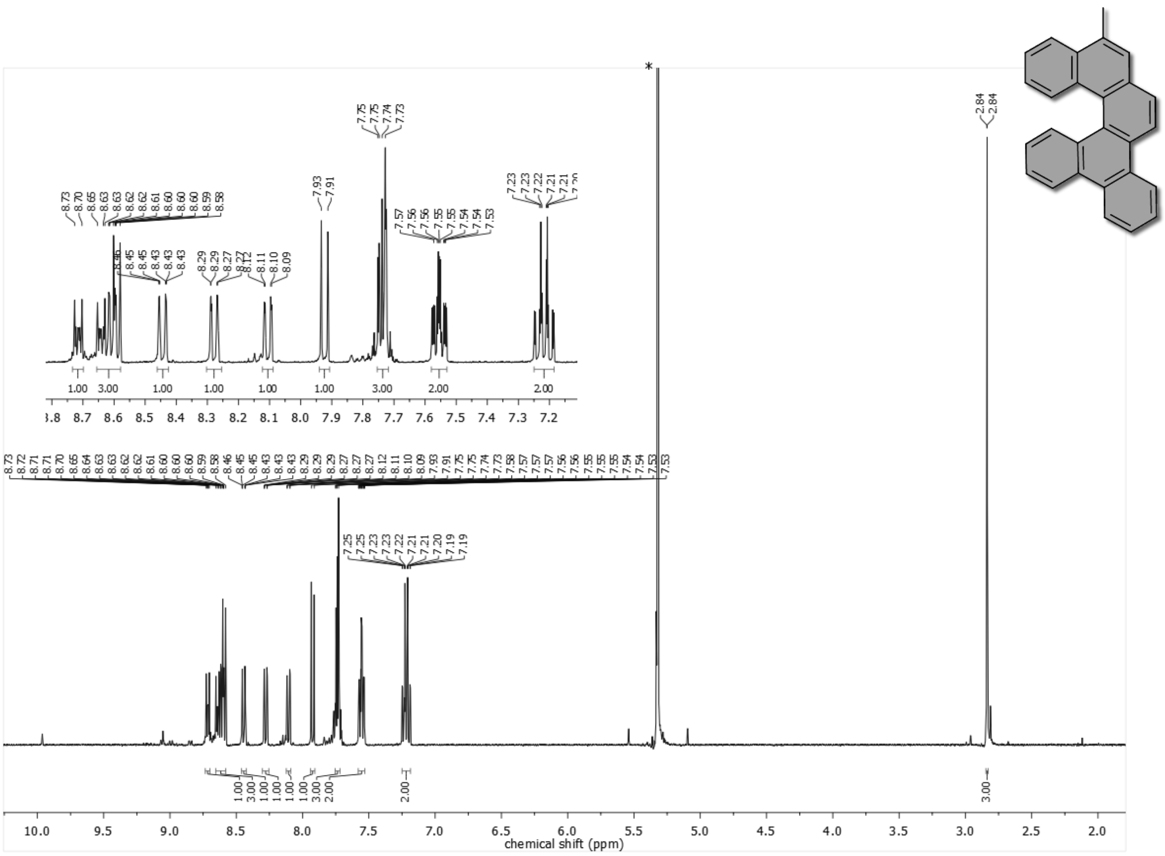

Supplementary Figure 23. 400MHz ^{1}H-NMR of 2-methyldibenzo[c,g]chrysene in CD_2Cl_2

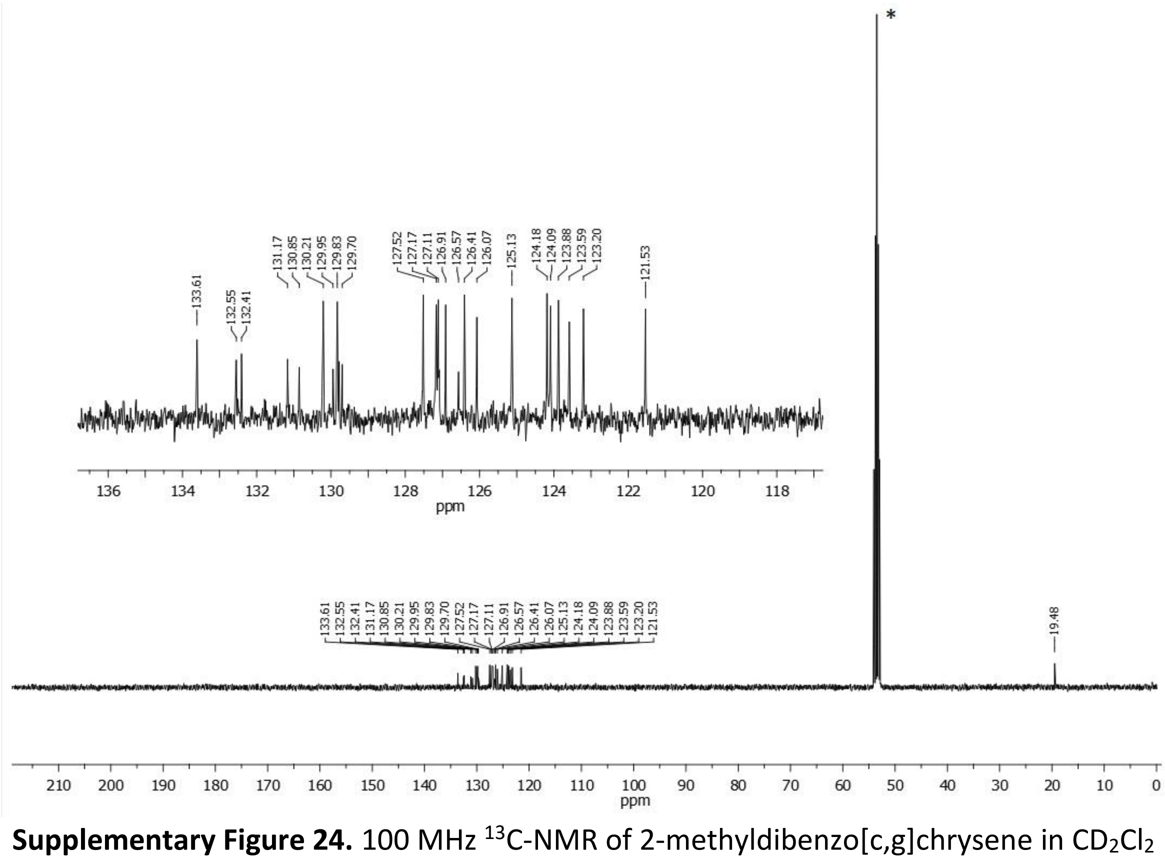

Supplementary Figure 24. 100 MHz ^{13}C-NMR of 2-methyldibenzo[c,g]chrysene in CD_2Cl_2

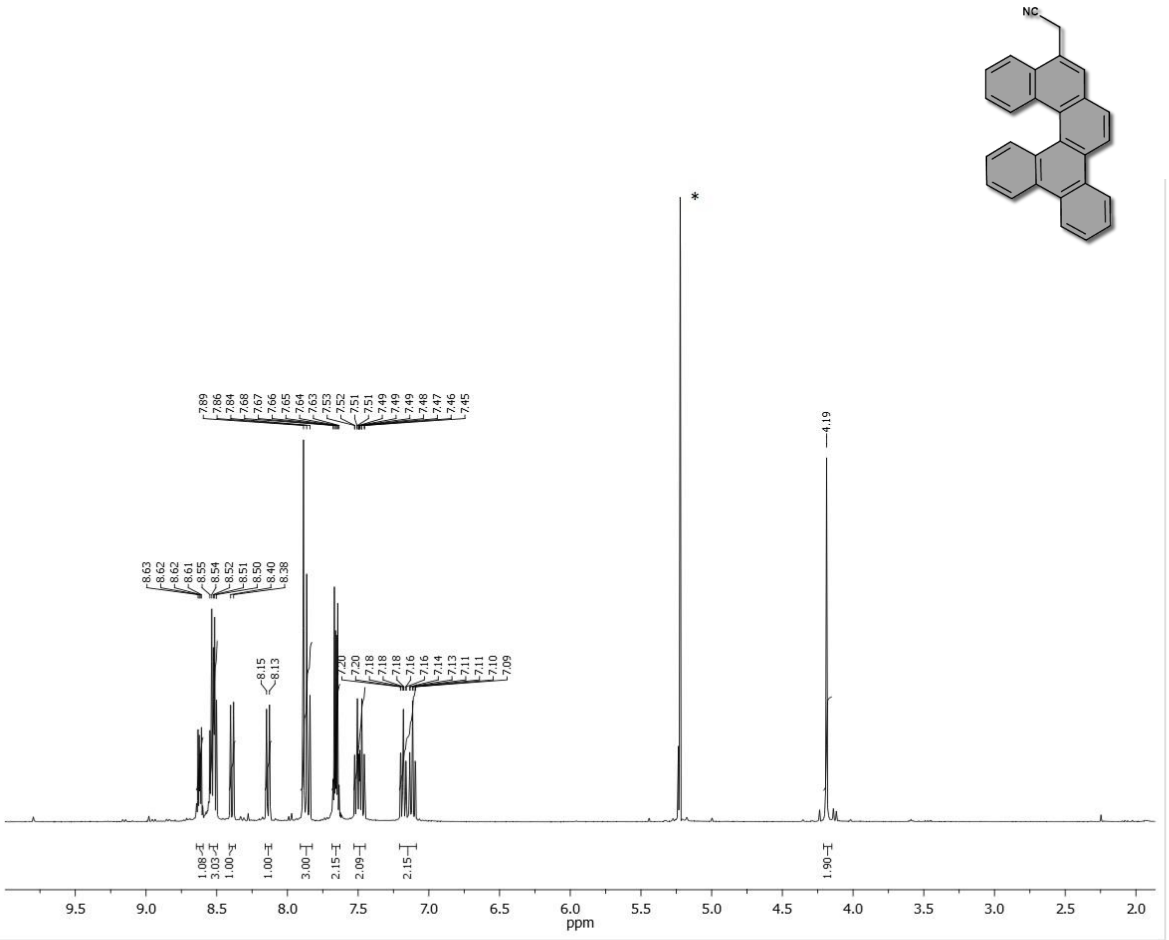

Supplementary Figure 25. 400 MHz ^{1}H-NMR of 2-(dibenzo[c,g]chrysen-2-yl)acetonitrile in CD_2Cl_2

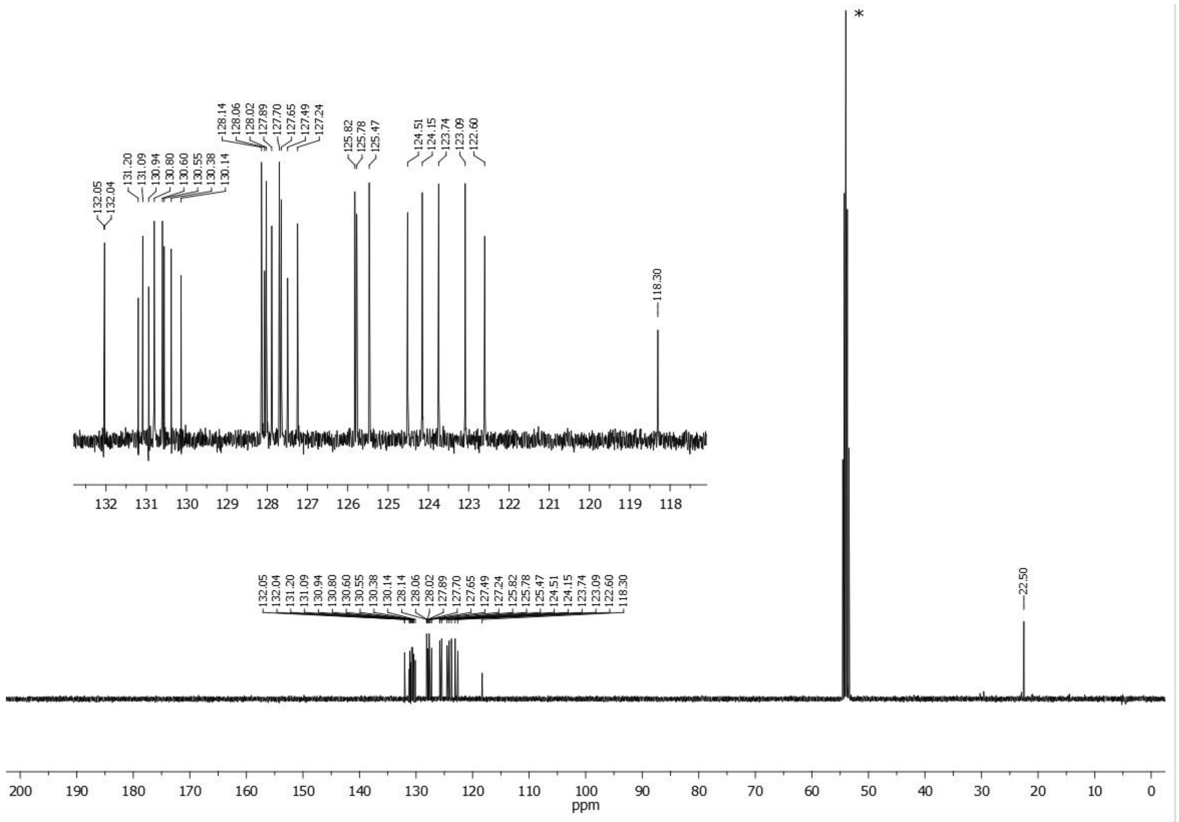

Supplementary Figure 26. 100 MHz ^{13}C-NMR of 2-(dibenzo[c,g]chrysen-2-yl)acetonitrile in CD_2Cl_2

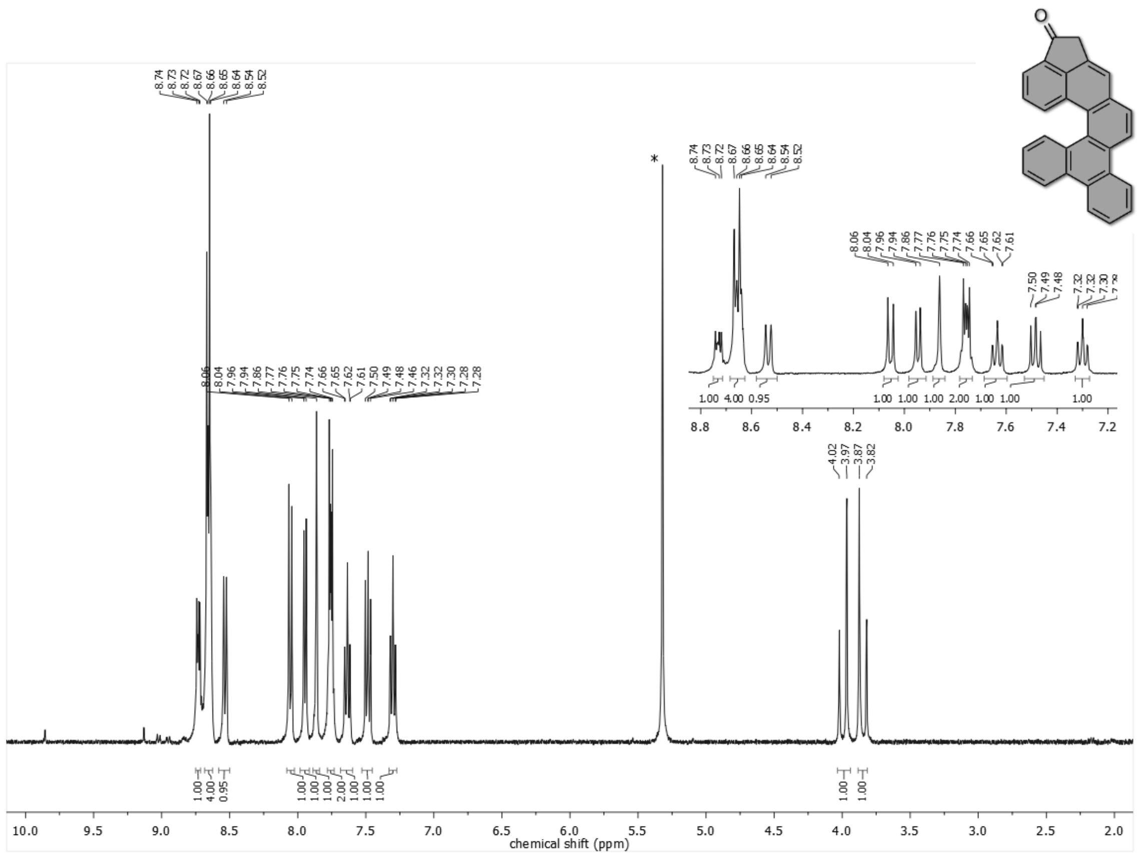

Supplementary Figure 27. 400 MHz ^{1}H-NMR of benzo[g]indeno[1,7-bc]chrysen-9(8H)-one in CD_2Cl_2

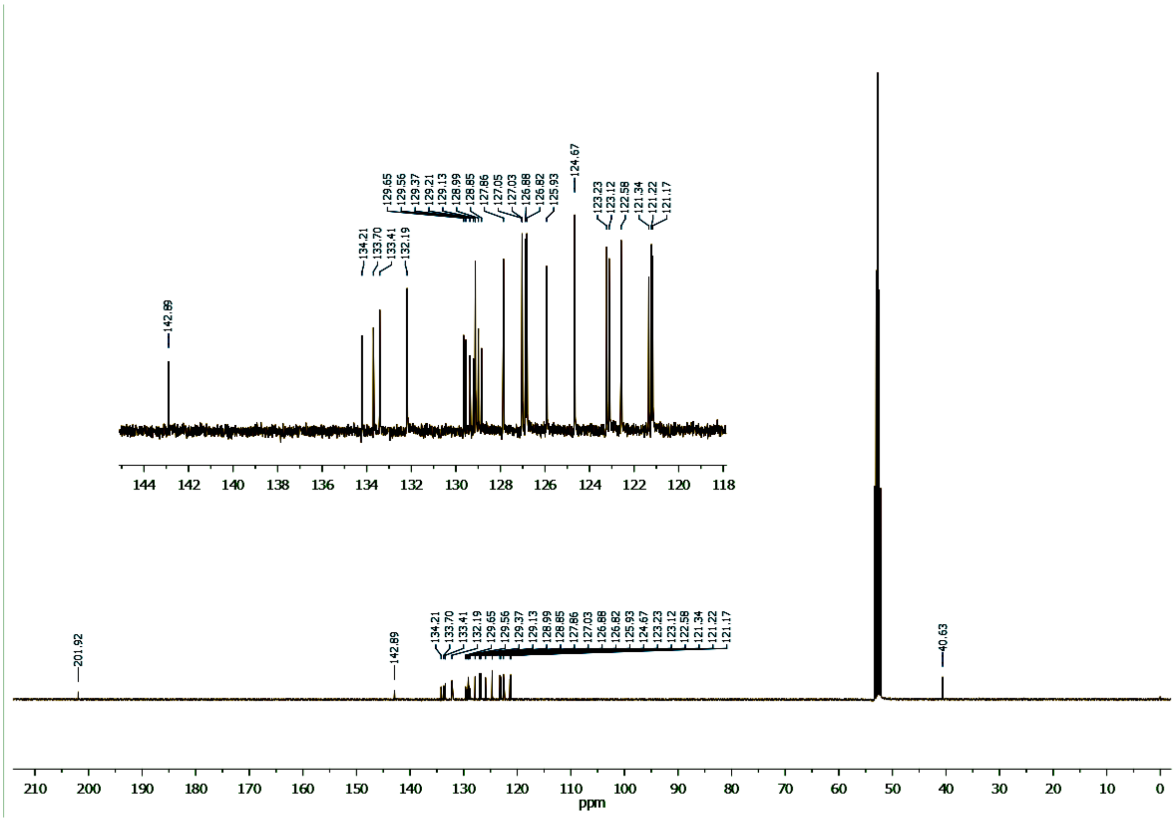

Supplementary Figure 28. 100 MHz ^{13}C-NMR of benzo[g]indeno[1,7-bc]chrysen-9(8H)-one in CD_2Cl_2

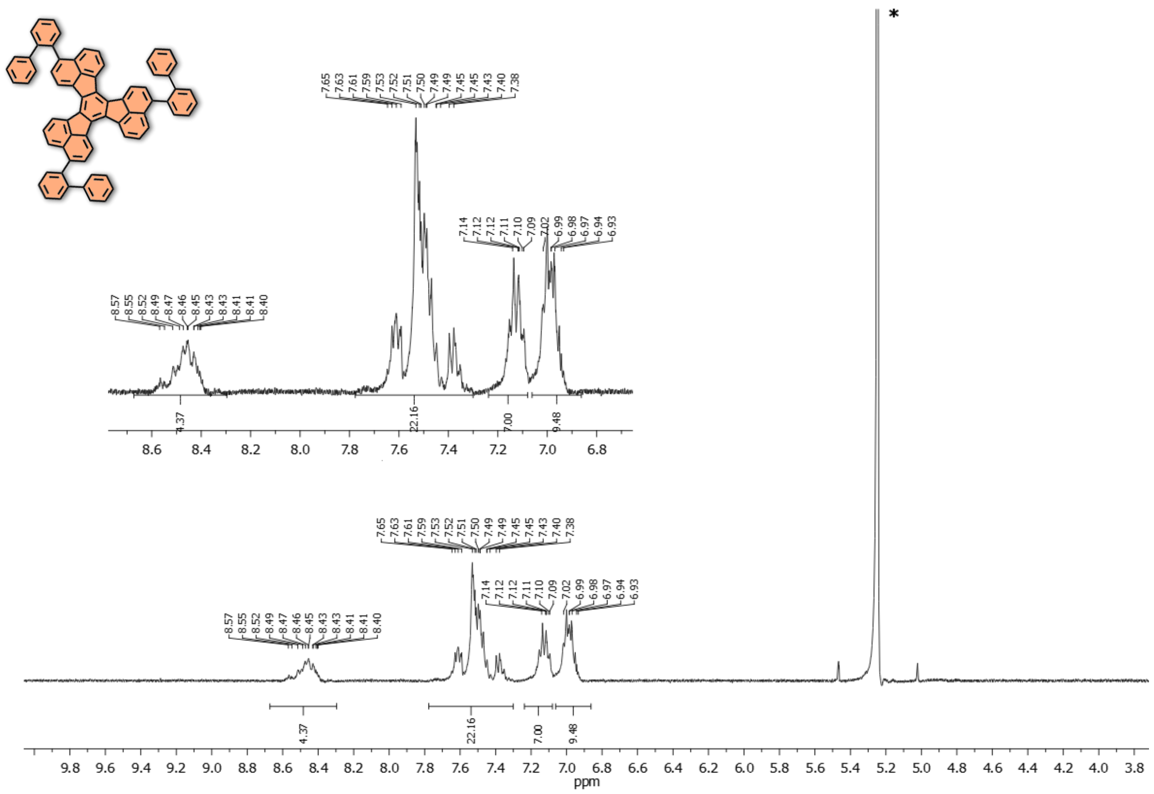

Supplementary Figure 29. 400 MHz ^{1}H-NMR of AAA in CD_2Cl_2

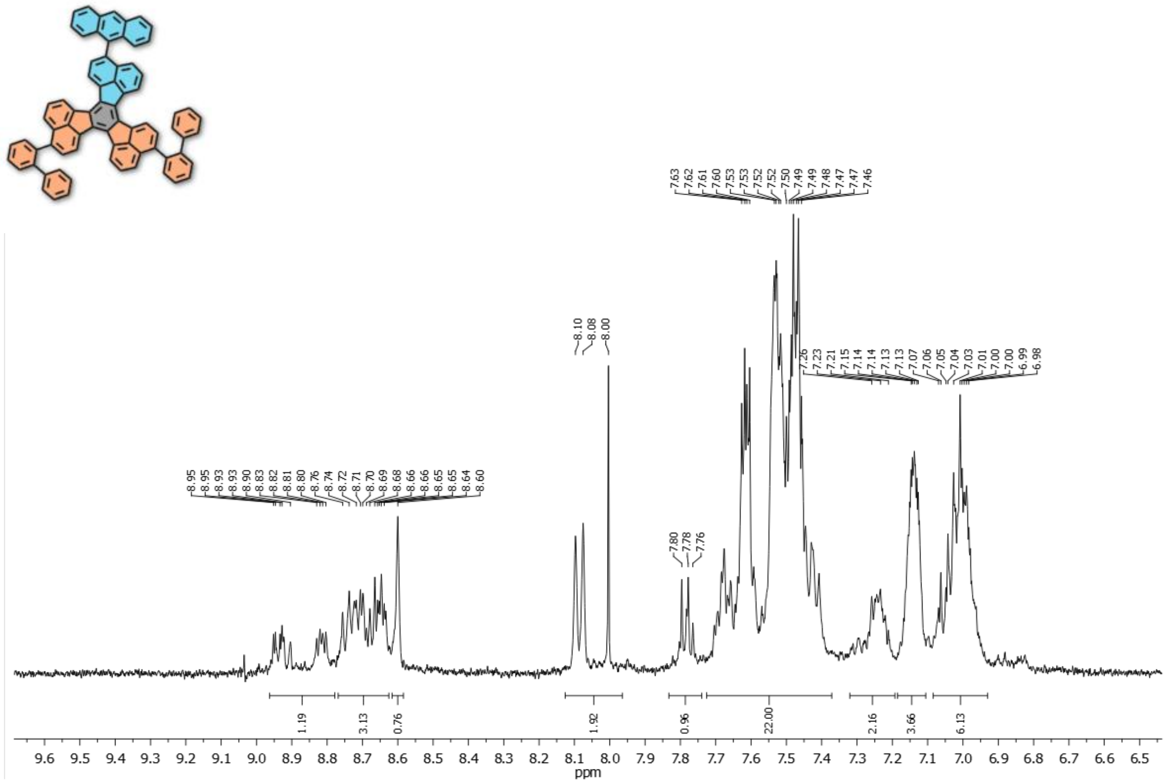

Supplementary Figure 30. 400 MHz ^{1}H-NMR of AAB in CD_2Cl_2

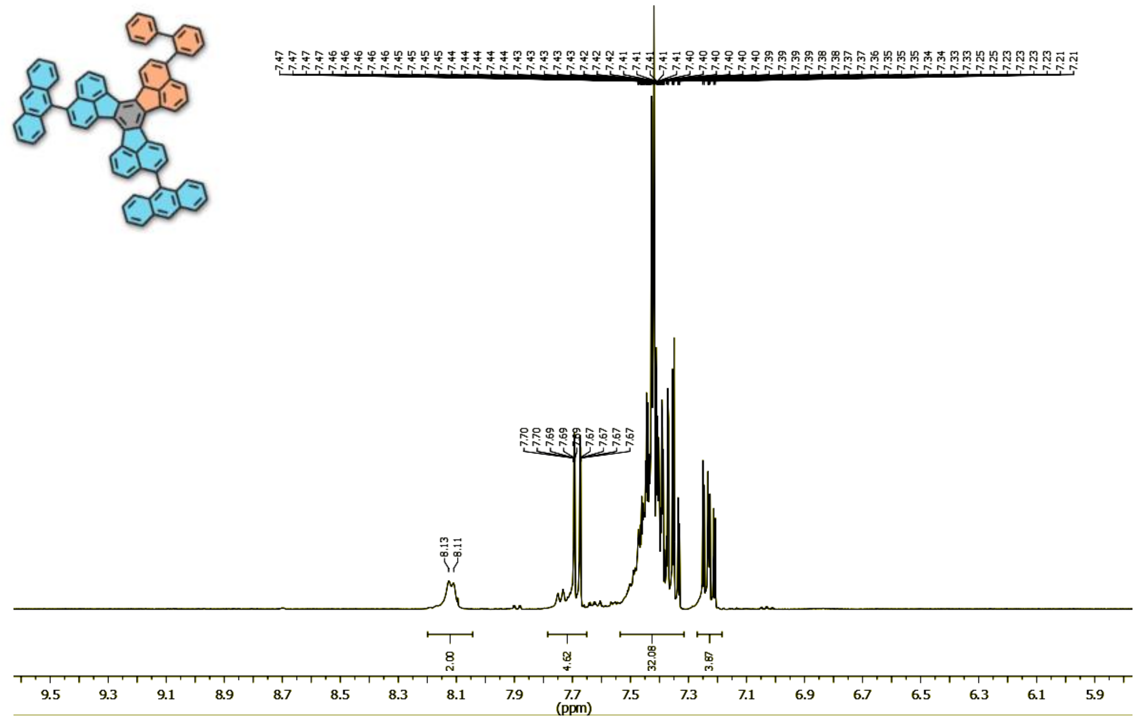

Supplementary Figure 31. 400 MHz ^{1}H-NMR of ABB in CD_2Cl_2

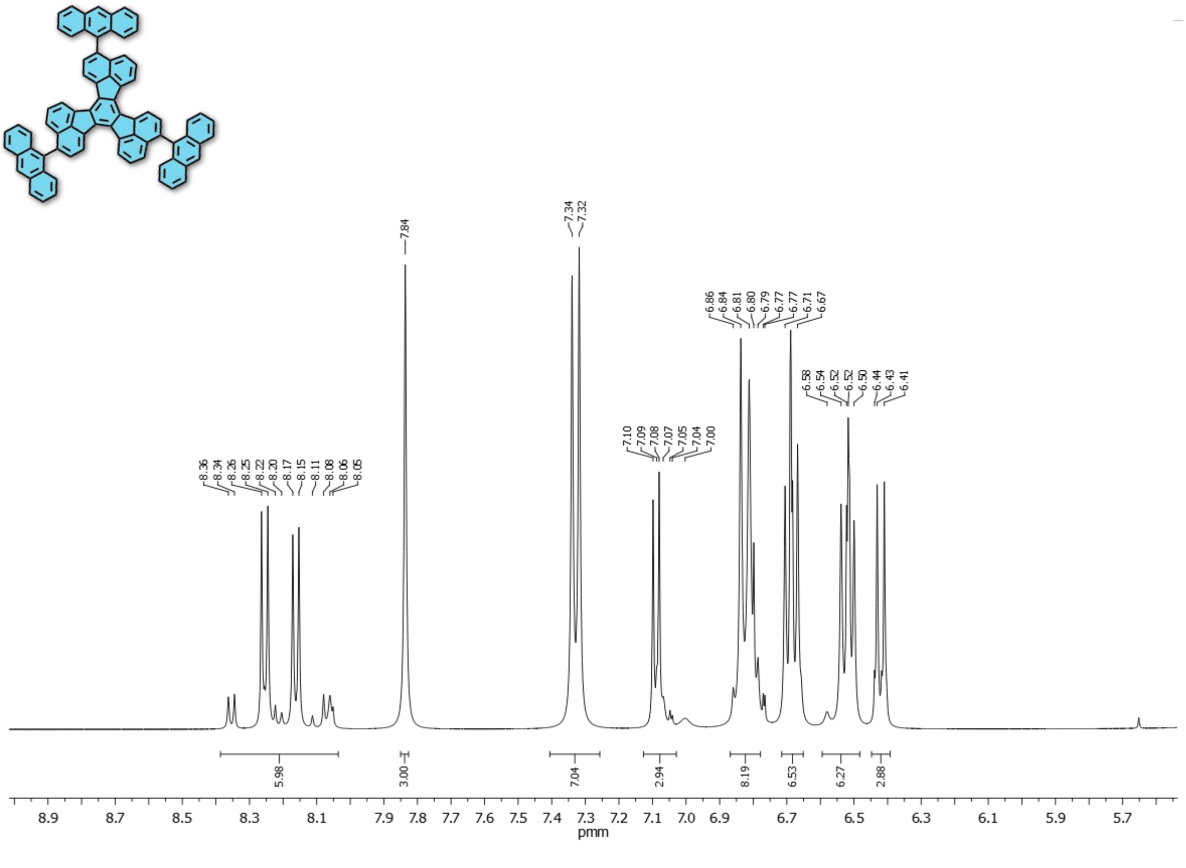

Supplementary Figure 32. 400 MHz ^{1}H-NMR of BBB in CD_2Cl_2

MS-Data (MALDI-TOF)

Tables containing the calculated exact masses and formulas of all combinations for tri-, tetra-, penta- and hexamers of the displayed segments A, B and C (combinations consisting of all three monomers are excluded).

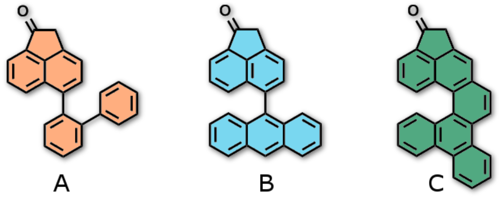

Supplementary Table 2. Segment combination, formula and exact mass

Trimers		
Segment combination	Formula	Exact Mass
**AAA**	$C_{72}H_{42}$	906.33
**AAB**	$C_{74}H_{42}$	930.33
**ABB**	$C_{76}H_{42}$	954.33
**BBB**	$C_{78}H_{42}$	978.33
**AAC**	$C_{76}H_{42}$	954.33
**ACC**	$C_{80}H_{42}$	1002.33
**BBC**	$C_{80}H_{42}$	1002.33
**BCC**	$C_{82}H_{42}$	1026.33
**CCC**	$C_{84}H_{42}$	1050.33

Tetramers		
Segment combination	Formula	Exact Mass
**AAAA**	$C_{96}H_{56}$	1208.44
**AAAB**	$C_{98}H_{56}$	1232.44
**AABB**	$C_{100}H_{56}$	1256.44
**ABBB**	$C_{102}H_{56}$	1280.44
**BBBB**	$C_{104}H_{56}$	1304.44
**AAAC**	$C_{100}H_{56}$	1256.44
**AACC**	$C_{104}H_{56}$	1304.44
**ACCC**	$C_{108}H_{56}$	1352.44
**CCCC**	$C_{112}H_{56}$	1400.44
**BBBC**	$C_{106}H_{56}$	1328.44
**BBCC**	$C_{108}H_{56}$	1352.44
**BCCC**	$C_{110}H_{56}$	1376.44

Pentamers		
Segment combination	Formula	Exact Mass
**AAAAA**	$C_{120}H_{70}$	1510.55
**AAAAB**	$C_{122}H_{70}$	1534.55
**AAABB**	$C_{124}H_{70}$	1558.55
**AABBB**	$C_{126}H_{70}$	1582.55
**ABBBB**	$C_{128}H_{70}$	1606.55
**BBBBB**	$C_{130}H_{70}$	1630.55
**AAAAC**	$C_{124}H_{70}$	1558.55
**AAACC**	$C_{128}H_{70}$	1606.55
**AACCC**	$C_{132}H_{70}$	1654.55
**ACCCC**	$C_{136}H_{70}$	1702.55
**CCCCC**	$C_{140}H_{70}$	1750.55
**BBBBC**	$C_{132}H_{70}$	1654.55
**BBBCC**	$C_{134}H_{70}$	1678.55
**BBCCC**	$C_{136}H_{70}$	1702.55
**BCCCC**	$C_{138}H_{70}$	1726.55

Hexamers		
Segment combination	Formula	Exact Mass
**AAAAAA**	$C_{144}H_{84}$	1812.66
**AAAAAB**	$C_{146}H_{84}$	1836.66
**AAAABB**	$C_{148}H_{84}$	1860.66
**AAABBB**	$C_{150}H_{84}$	1884.66
**AABBBB**	$C_{152}H_{84}$	1908.66
**ABBBBB**	$C_{154}H_{84}$	1932.66
**BBBBBB**	$C_{156}H_{84}$	1956.66
**AAAAAC**	$C_{148}H_{84}$	1860.66
**AAAACC**	$C_{152}H_{84}$	1908.66
**AAACCC**	$C_{156}H_{84}$	1956.66
**AACCCC**	$C_{160}H_{84}$	2004.66
**ACCCCC**	$C_{164}H_{84}$	2052.66
**CCCCCC**	$C_{168}H_{84}$	2100.66
**BBBBBC**	$C_{158}H_{84}$	1980.66
**BBBBCC**	$C_{160}H_{84}$	2004.66
**BBBCCC**	$C_{162}H_{84}$	2028.66
**BBCCCC**	$C_{164}H_{84}$	2052.66
**BCCCCC**	$C_{166}H_{84}$	2076.66

Combination of the segments A and B

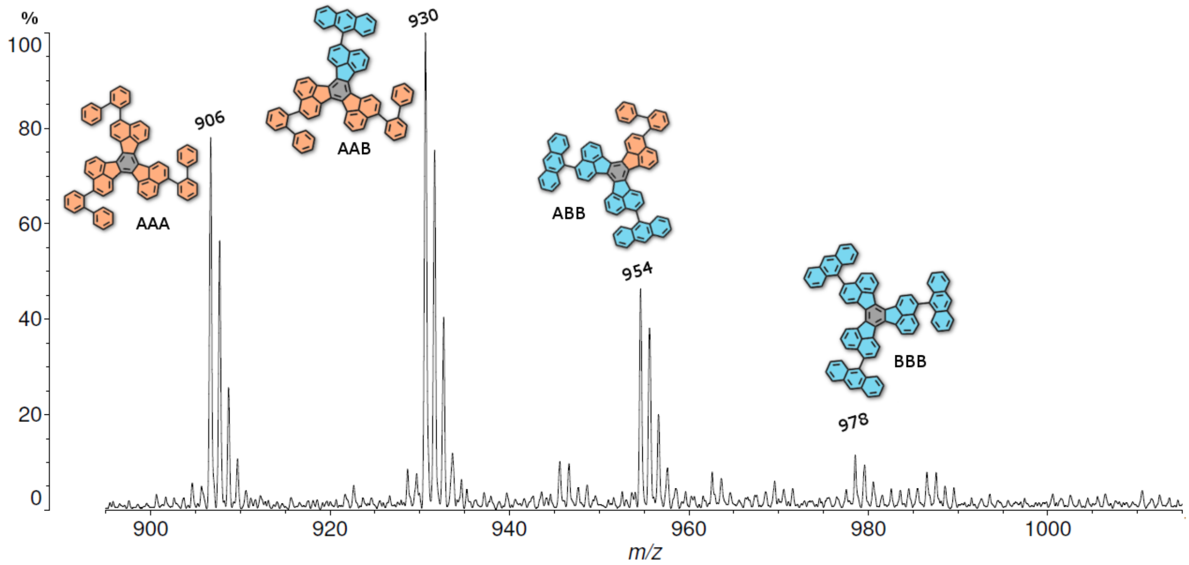

Supplementary Figure 33. - MS-Spectrum of the reaction mixture after cyclomerization of **A** and **B**

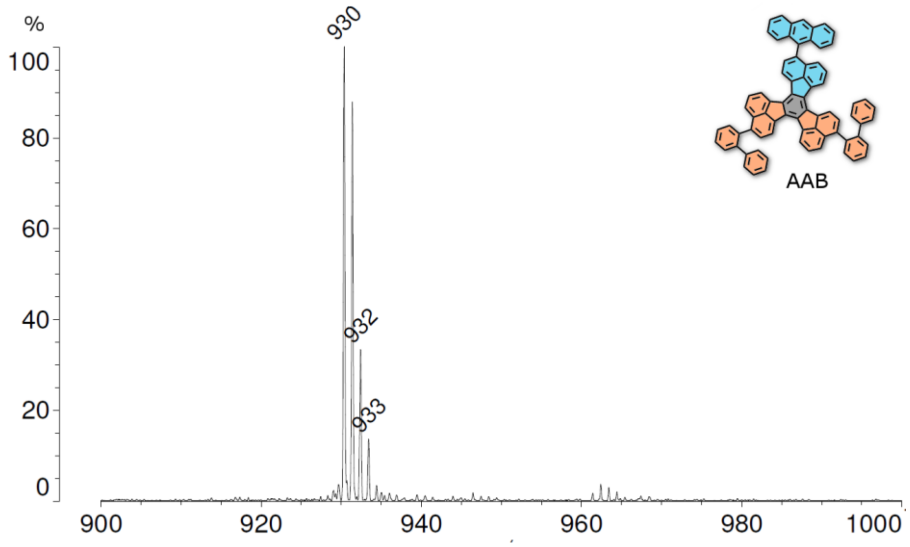

Supplementary Figure 34. - MS-Spectrum of the trimer **AAB** after HPLC separation

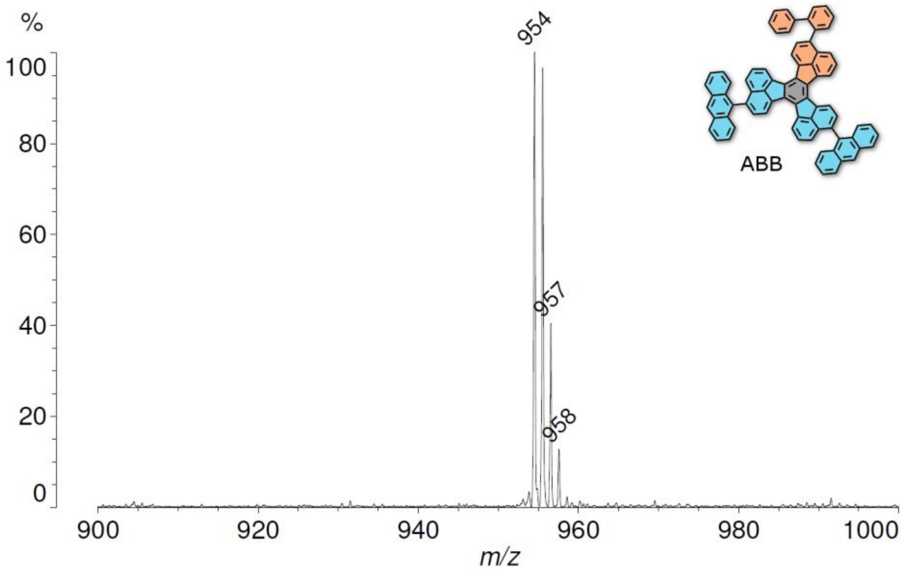

Supplementary Figure 35. - MS-Spectrum of the trimer **ABB** after HPLC separation

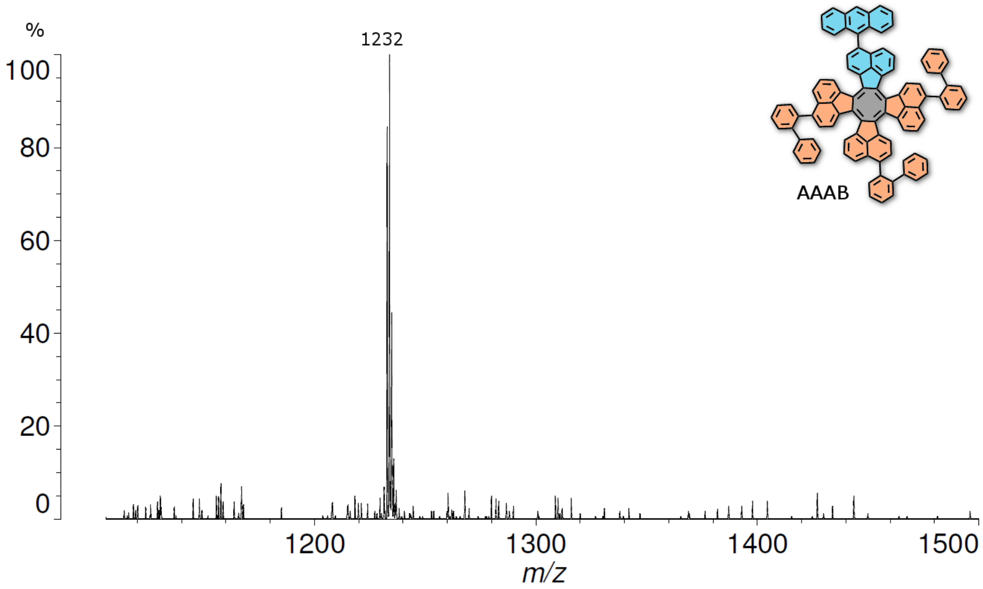

Supplementary Figure 36. - MS-Spectrum of the tetramer **AAAB** after analytical HPLC separation

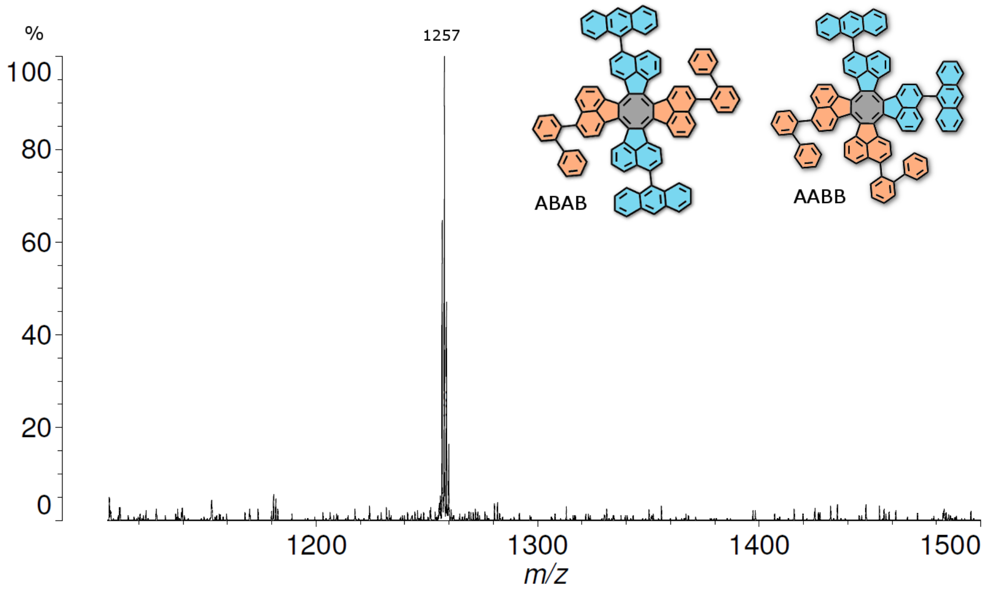

Supplementary Figure 37. - MS-Spectrum of **AABB** and **ABAB** after analytical HPLC separation

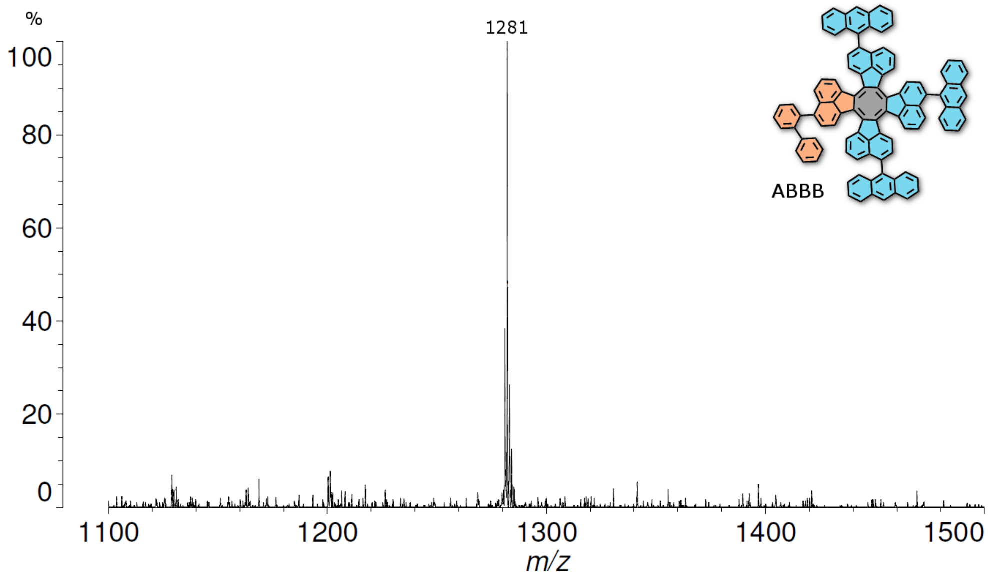

Supplementary Figure 38. - MS-Spectrum of the tetramer **ABBB** after analytical HPLC separation

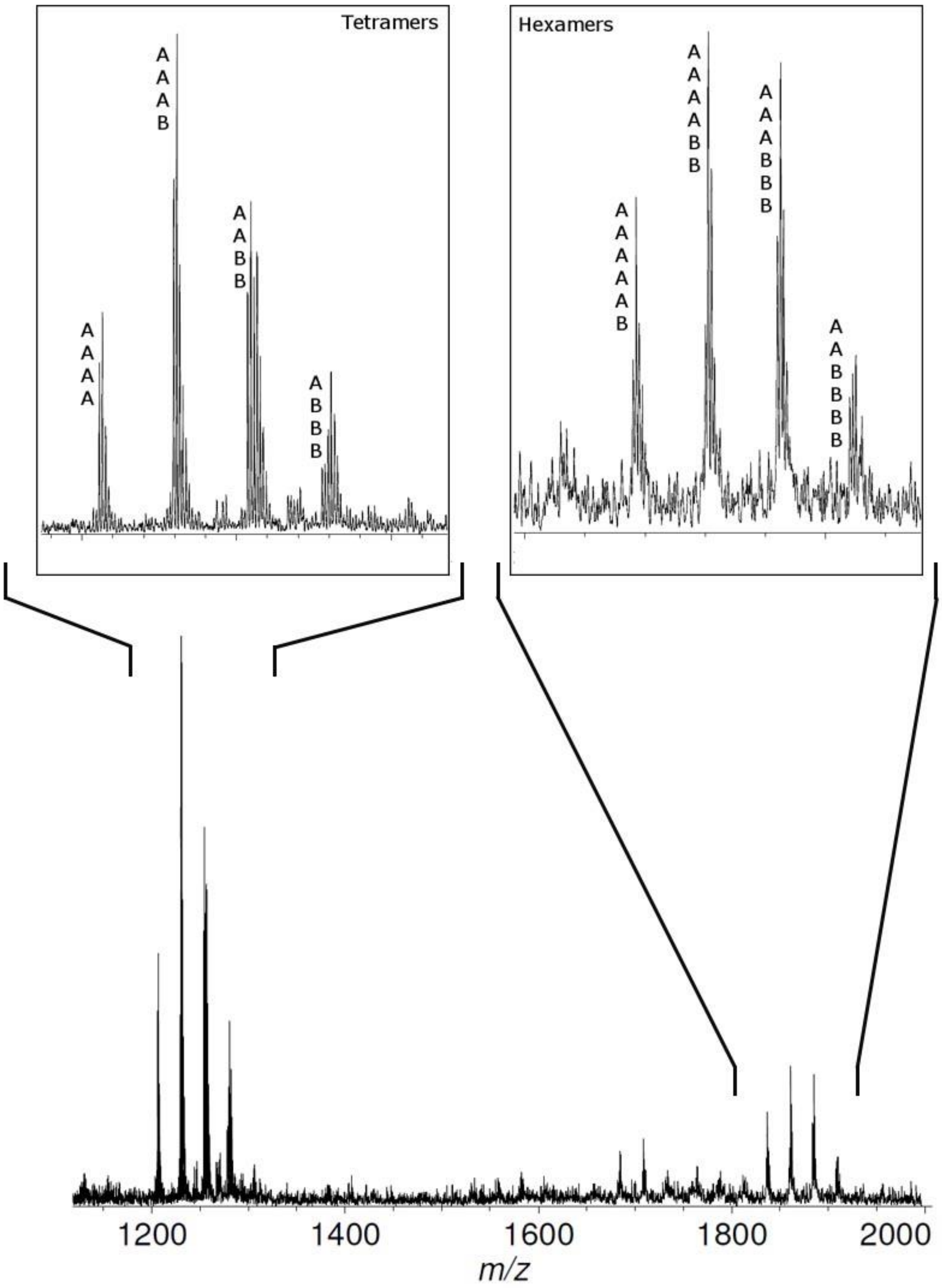

Supplementary Figure 39. - Extended MS-Spectrum of the combination of segment **A** and **B**

Combination of the segments A and C

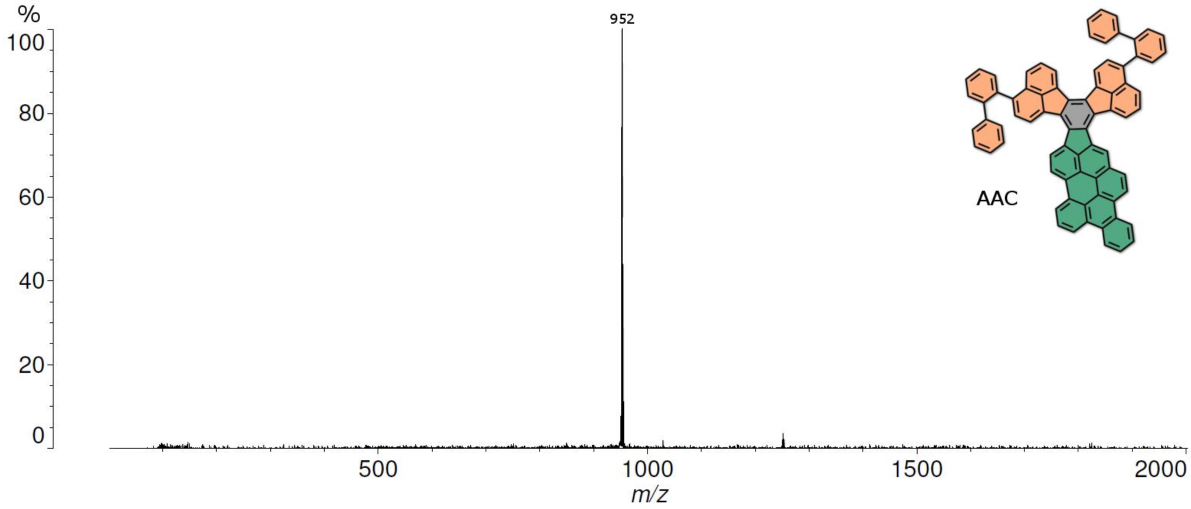

Supplementary Figure 40. - MS-Spectrum of **AAC** with closed **C**-segment, after HPLC separation

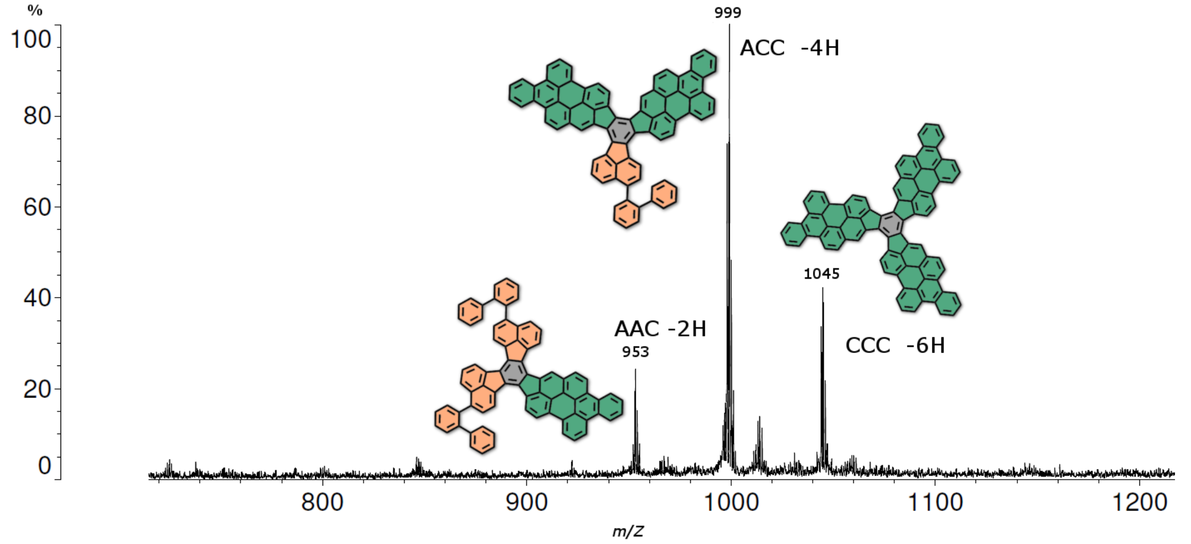

Supplementary Figure 41. - MS-Spectrum of the insoluble trimers **ACC** and **CCC**, plus soluble trimer **AAC**

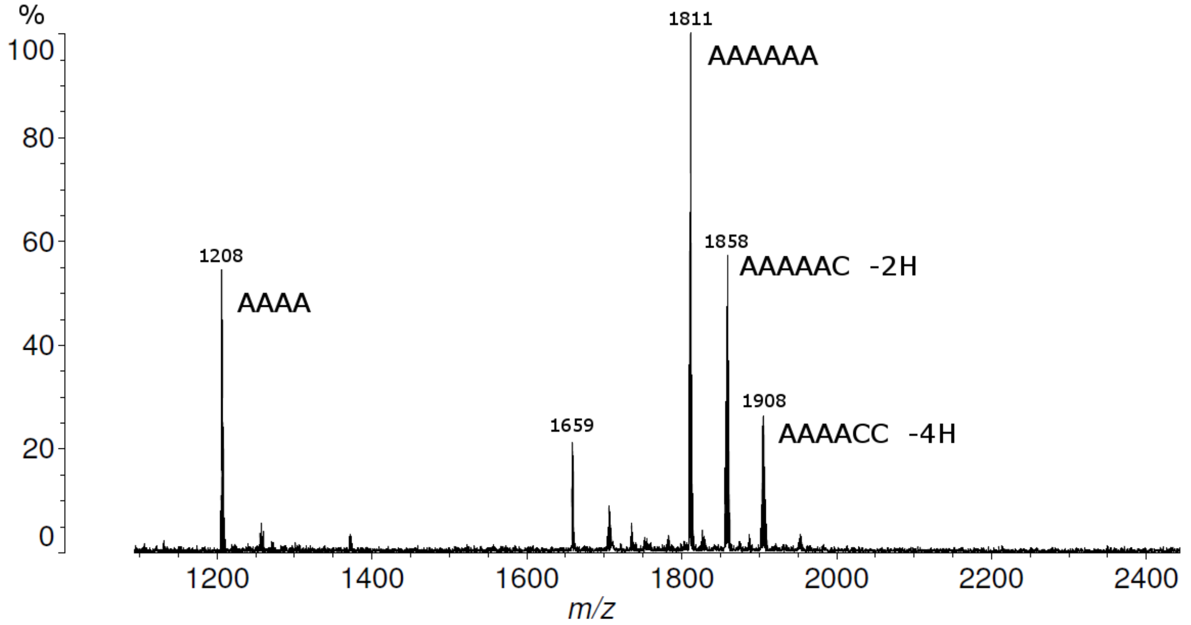

Supplementary Figure 42. - MS-Spectrum of fraction containing the soluble tetra- and hexamers

Combination of the segments B and C

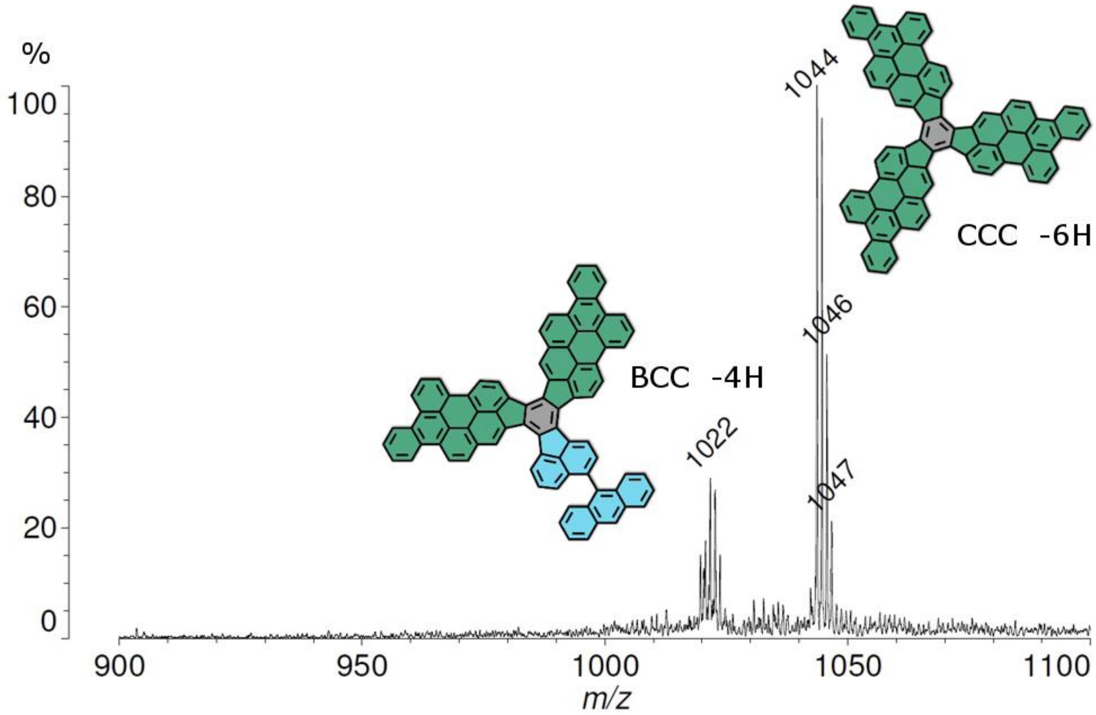

Supplementary Figure 43. - MS-Spectrum of the insoluble trimers **BCC** and **CCC** with a closed **C**-segment.

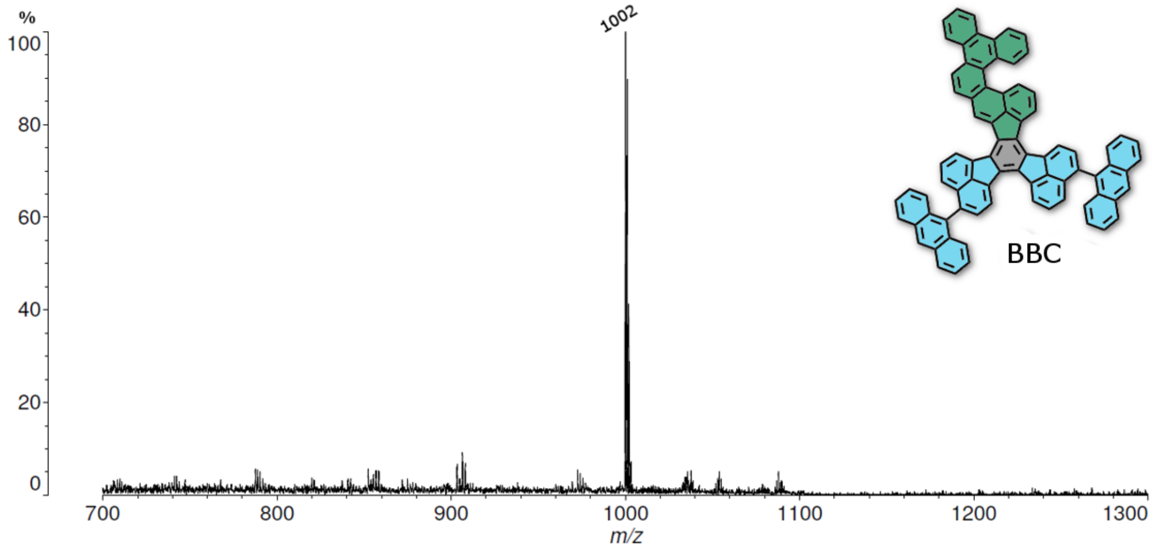

Supplementary Figure 44. - MS-Spectrum of the trimer **BBC**

Cyclomerization of segment A

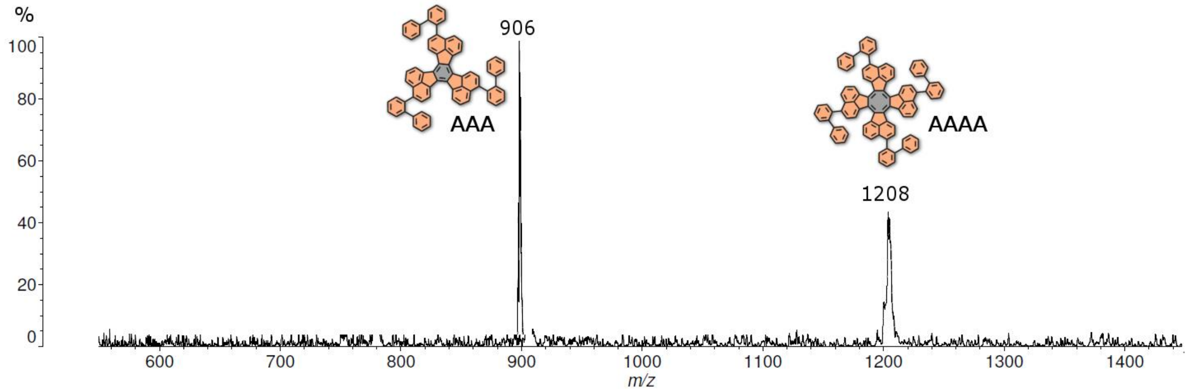

Supplementary Figure 45. - MS-Spectrum of the reaction mixture of the cyclomerization of segment A after silica plug with toluene.

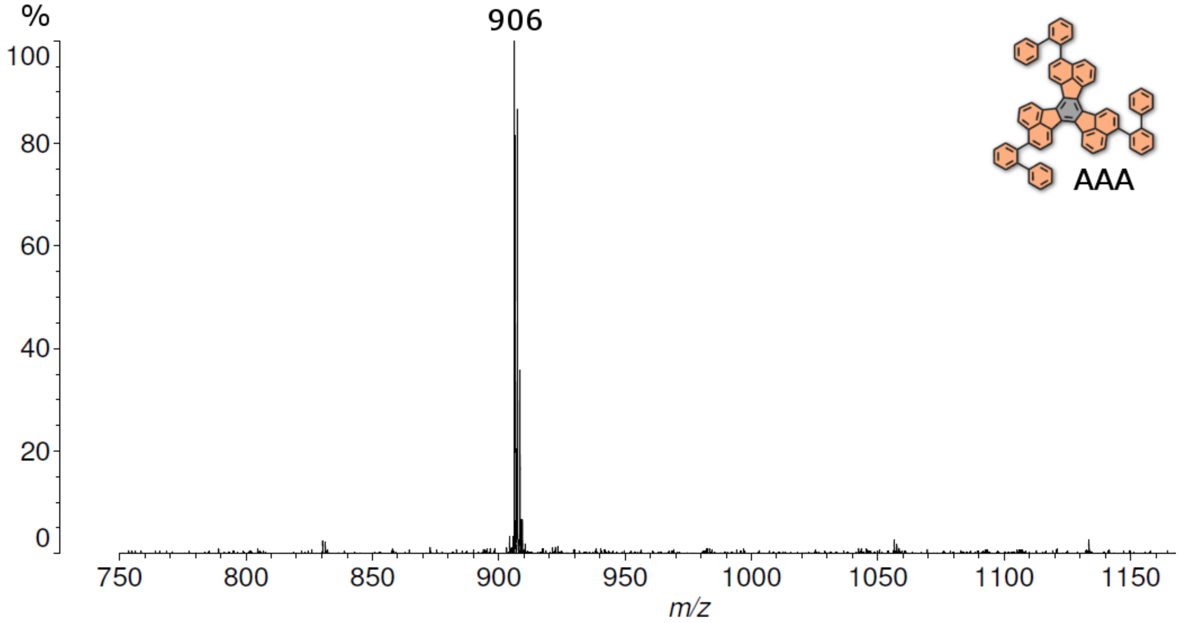

Supplementary Figure 46. - MS-Spectrum of trimer **AAA** after HPLC separation

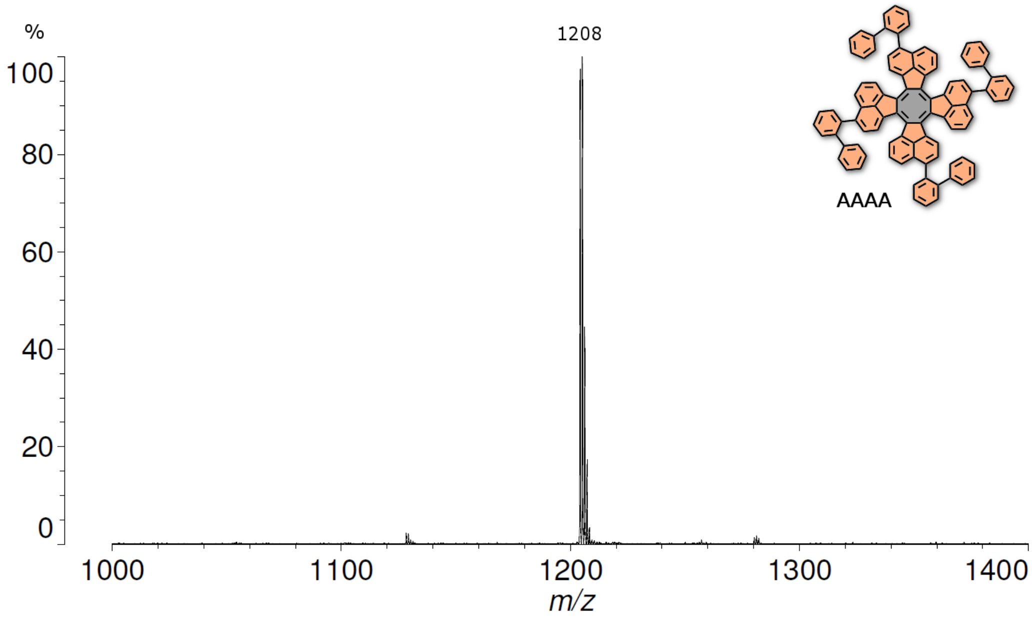

Supplementary Figure 47. - MS-Spectrum of trimer **AAAA** after HPLC separation

Cyclomerization of segment B

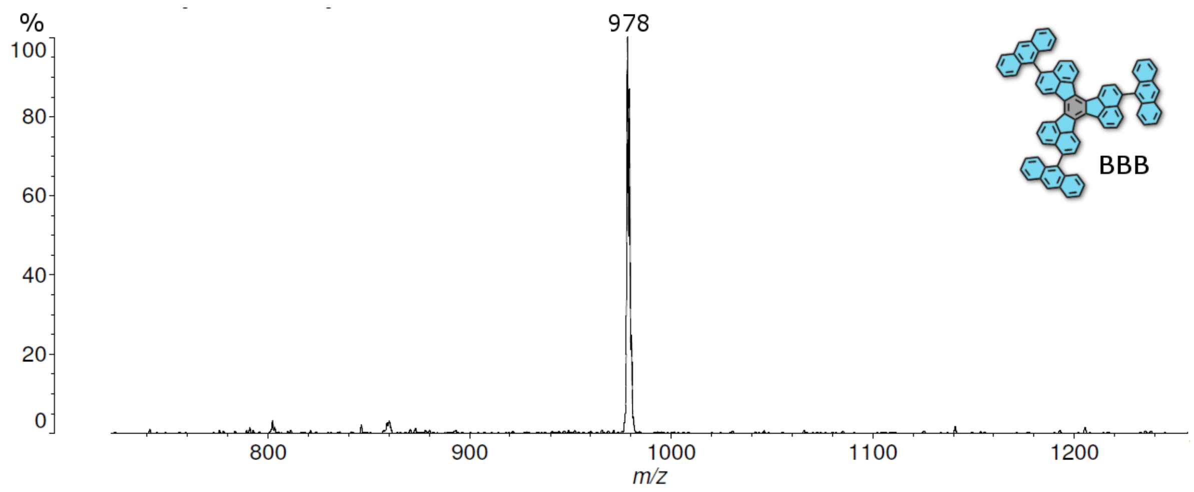

Supplementary Figure 48. - MS-Spectrum of trimer **BBB** after HPLC separation

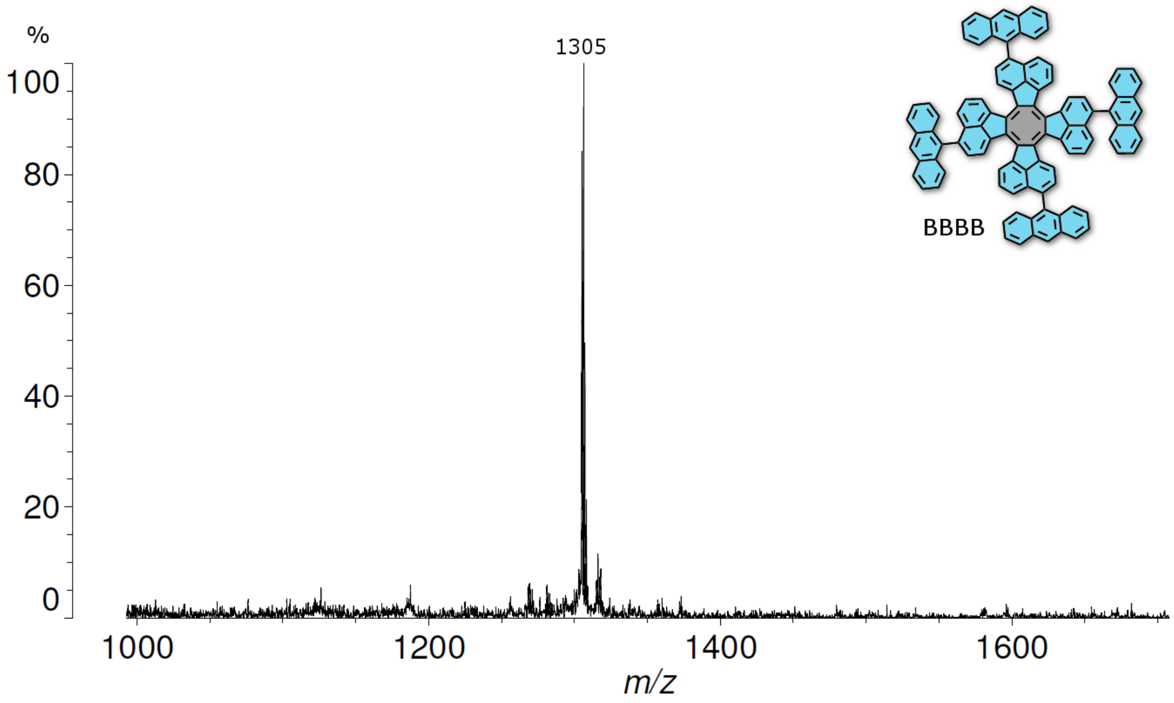

Supplementary Figure 49. - MS-Spectrum of trimer **BBBB** after analytical HPLC separation

Crystallographic Data

Segment A - (S9)

CCDC no.	1858611
Empirical formula	$C_{24}H_{16}O$
Formula weight	320.37
Temperature/K	153.00(10)
Crystal system	monoclinic
Space group	$P2_1/c$
a/Å	12.1481(3)
b/Å	12.2294(3)
c/Å	11.6042(4)
α/°	90
β/°	101.787(3)
γ/°	90
Volume/Å^3	1687.62(9)
Z	4
ρ_{calc}g/cm^3	1.261
μ/mm^{-1}	0.586
F(000)	672.0
Crystal size/mm^3	0.115 × 0.102 × 0.075
Radiation	CuKα (λ = 1.54184)
2Θ range for data collection/°	7.434 to 113.348
Index ranges	-11 ≤ h ≤ 13, -12 ≤ k ≤ 13, -12 ≤ l ≤ 11
Reflections collected	7078
Independent reflections	2205 [R_{int} = 0.0322, R_{sigma} = 0.0252]
Data/restraints/parameters	2205/142/452
Goodness-of-fit on F^2	1.052
Final R indexes [I>=2σ (I)]	R_1 = 0.0414, wR_2 = 0.1111
Final R indexes [all data]	R_1 = 0.0559, wR_2 = 0.1239
Largest diff. peak/hole / e Å^{-3}	0.20/-0.11
Goniometer type:	Agilent SuperNova with Atlas detector
Structure solution :	SHELXS-2014
Structure refinement :	SHELXL-2014
Hydrogen treatment:	riding model
Disorder:	Disoder in two molecule layers: 0.67 : 0.33% occupation
Comments:	Refinement of disorder of the whole structure with DELU command (rigid bond) for providing stabilisation of the disordered structure.

Segment A´ - (S16)

CCDC no.	1858612
Empirical formula	$C_{24}H_{14}O$
Formula weight	318.35
Temperature/K	152.95(10)
Crystal system	orthorhombic
Space group	$P2_12_12_1$
a/Å	16.6981(7)
b/Å	12.5257(6)
c/Å	7.2075(2)
α/°	90
β/°	90
γ/°	90
Volume/Å^3	1507.49(10)
Z	4
ρ_{calc}g/cm^3	1.403
μ/mm^{-1}	0.656
F(000)	664.0
Crystal size/mm^3	0.302 × 0.06 × 0.048
Radiation	CuKα (λ = 1.54184)
2Θ range for data collection/°	8.826 to 117.898
Index ranges	$-18 \leq h \leq 18$, $-13 \leq k \leq 12$, $-7 \leq l \leq 5$
Reflections collected	6507
Independent reflections	2122 [R_{int} = 0.0661, R_{sigma} = 0.0523]
Data/restraints/parameters	2122/121/276
Goodness-of-fit on F^2	1.070
Final R indexes [I>=2σ (I)]	R_1 = 0.0812, wR_2 = 0.1823
Final R indexes [all data]	R_1 = 0.1103, wR_2 = 0.1995
Largest diff. peak/hole / e Å^{-3}	0.19/-0.17
Flack parameter	-0.3(19)
Goniometer type:	Agilent SuperNova with Atlas detector
Structure solution :	SHELXS-2014
Structure refinement :	SHELXL-2014
Hydrogen treatment:	riding model
Disorder:	Whole molecule with 57:43% occupation
Comments:	reciprocal space/lattice showed at least one twin component, which was excluded as much as possible for refinement weaker data set<br>absolute structure could not get determined because of disorder and twinning situation; racemic twin refinement was implemented: BASF = -0.3(19)

Segment B - (S8)

CCDC no.	1858613
Empirical formula	$C_{26}H_{16}O$
Formula weight	344.39
Temperature/K	156(6)
Crystal system	monoclinic
Space group	$P2_1/c$
a/Å	11.6384(4)
b/Å	15.3006(4)
c/Å	20.0485(8)
α/°	90
β/°	101.959(4)
γ/°	90
Volume/Å^3	3492.6(2)
Z	8
ρ_{calc}g/cm^3	1.310
μ/mm^{-1}	0.607
F(000)	1440.0
Crystal size/mm^3	0.267 × 0.124 × 0.115
Radiation	CuKα (λ = 1.54184)
2Θ range for data collection/°	7.328 to 126.53
Index ranges	$-13 \le h \le 13, -17 \le k \le 16, -22 \le l \le 10$
Reflections collected	8565
Independent reflections	5433 [R_{int} = 0.0205, R_{sigma} = 0.0357]
Data/restraints/parameters	5433/0/487
Goodness-of-fit on F^2	1.018
Final R indexes [I>=2σ (I)]	R_1 = 0.0532, wR_2 = 0.1340
Final R indexes [all data]	R_1 = 0.0731, wR_2 = 0.1522
Largest diff. peak/hole / e Å^{-3}	0.64/-0.28
Goniometer type:	Agilent SuperNova with Atlas detector
Structure solution :	SHELXS-2014
Structure refinement :	SHELXL-2014
Hydrogen treatment:	riding model
Disorder:	-
Comments:	2 independent molecules in asym. unit

Segment C - (S22)

CCDC no.	1858610
Empirical formula	$C_{28}H_{16}O$
Formula weight	368.41
Temperature/K	156(5)
Crystal system	orthorhombic
Space group	$P2_12_12_1$
a/Å	11.2608(4)
b/Å	13.3385(6)
c/Å	23.3712(10)
α/°	90
β/°	90
γ/°	90
Volume/Å^3	3510.4(2)
Z	8
ρ_{calc}g/cm^3	1.394
μ/mm^{-1}	0.645
F(000)	1536.0
Crystal size/mm^3	0.375 × 0.261 × 0.104
Radiation	CuKα (λ = 1.54184)
2Θ range for data collection/°	7.566 to 123.408
Index ranges	-12 ≤ h ≤ 8, -10 ≤ k ≤ 14, -26 ≤ l ≤ 19
Reflections collected	8363
Independent reflections	5268 [R_{int} = 0.0413, R_{sigma} = 0.0600]
Data/restraints/parameters	5268/0/524
Goodness-of-fit on F^2	1.052
Final R indexes [I>=2σ (I)]	R_1 = 0.0495, wR_2 = 0.1197
Final R indexes [all data]	R_1 = 0.0599, wR_2 = 0.1325
Largest diff. peak/hole / e Å^{-3}	0.20/-0.24
Flack parameter	-0.4(7)
Goniometer type:	Agilent SuperNova with Atlas detector
Structure solution :	SHELXS-2014
Structure refinement :	SHELXL-2014
Hydrogen treatment:	riding model
Disorder:	-
Comments:	Refined as racemic Twin:<br>BASF 0.4(7)<br>2 independent molecules

UV/VIS Data

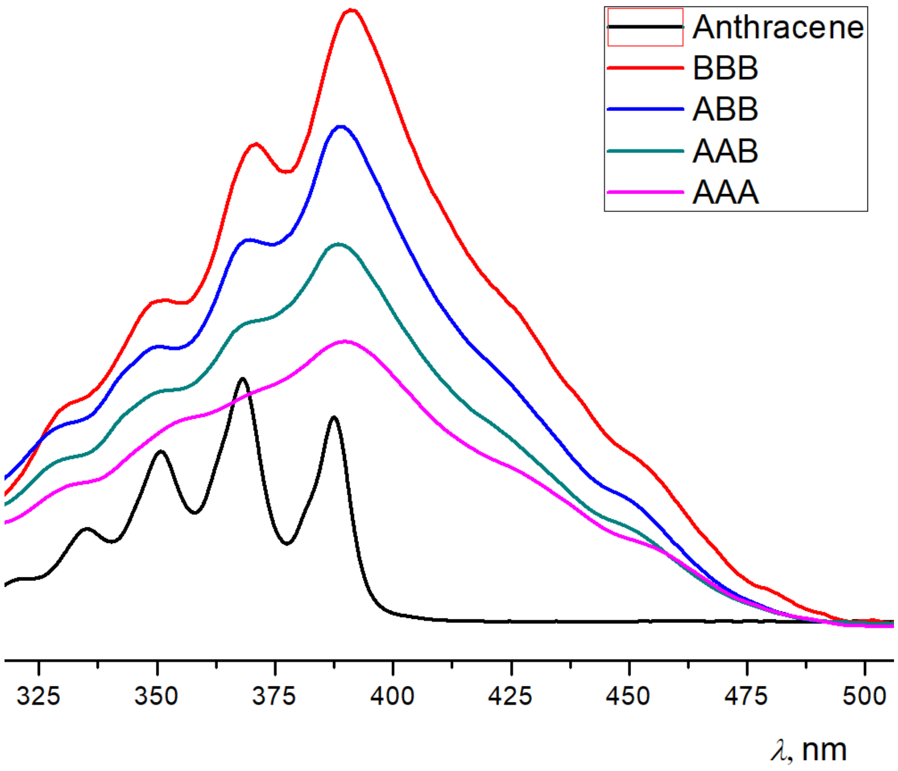

Supplementary Figure 50. UV/VIS Spectra of the trimers **AAA**, **AAB**, **ABB** & **BBB** compared to the UV/VIS of Anthracene, the shown intensities are relative.

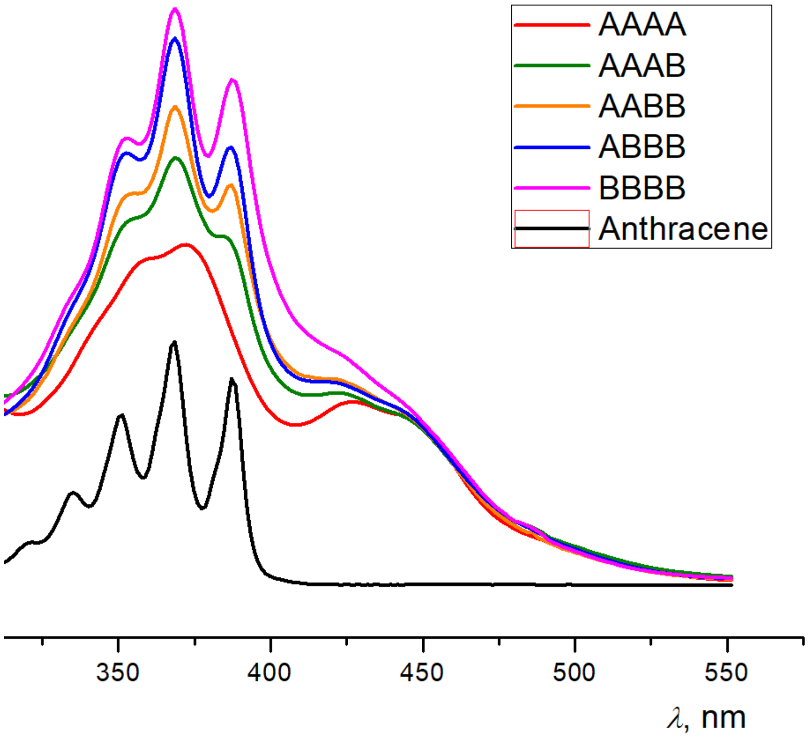

Supplementary Figure 51. UV/VIS Spectra of the tetramers **AAAA**, **AAAB**, **A2B2**, **ABBB** & **BBBB** compared to the UV/VIS of Anthracene, the shown intensities are relative.

Supplementary Table 3. SWCNT precursors obtained in this work

N	Segment combination	Chirality of the SWCNT seed (n,m)	Prepared in this work	Obtained in pure form
1	**AAA**	6,6	*	*
2	**AAB**	7,5	*	*
3	**ABB**	8,4	*	*
4	**BBB**	9,3	*	*
5	**AAC**	7,4	*	*
6	**ACC**	8,2	*	
7	**BBC**	9,2	*	*
8	**BCC**	9,1	*	
9	**CCC**	9,0	*	
10	**ABC**	8,3		
11	**AAAA**	8,8	*	*
12	**AAAB**	9,7	*	*
13	**AABB**	10,6	*	
14	**ABBB**	11,5	*	*
15	**BBBB**	12,4	*	*
16	**AAAC**	9,6	*	
17	**AACC**	10,4	*	
18	**ACCC**	11,2	*	
19	**CCCC**	12,0	*	
20	**BBBC**	12,3	*	
21	**BBCC**	12,2	*	
22	**BCCC**	12,1	*	
23	**AABC**	10,5		
24	**ABBC**	11,4		
25	**ABCC**	11,3		
